# Isolation of a Melanoblast Stimulator from *Dimocarpus longan*, Its Structural Modification, and Structure–Activity Relationships for Vitiligo

**DOI:** 10.3390/molecules27072135

**Published:** 2022-03-25

**Authors:** Jae-Won Song, Sunju Choi, Gayeong Kim, Hyang Bok Lee, P. Sankara Rao, Jeonghyun Shin, Eun Ki Kim, Dong-Gyu Cho

**Affiliations:** 1 Department of Chemistry and Chemical Engineering, Inha University, Incheon 22212, Korea; qkwldpdy@naver.com (J.-W.S.); p.sankarmsc89@gmail.com (P.S.R.); 2 Department of Biological Engineering, Inha University, Incheon 22212, Korea; tjswn1174@naver.com (S.C.); teclra94@naver.com (G.K.); hbjbb@hanmail.net (H.B.L.); ekkim@inha.ac.kr (E.K.K.); 3 Department of Dermatology, School of Medicine, Inha University, Incheon 22212, Korea; jshin@inha.ac.kr

**Keywords:** melanoblast, stimulator, melanogenesis, vitiligo structure–activity relationships

## Abstract

A novel melanoblast stimulator (**1**) was isolated from *Dimocarpus longan*. Its analogs were also synthesized to support a new furan-based melanoblast stimulator scaffold for treating vitiligo. Isolated 5-(hydroxymethyl)furfural (HMF, **1**) is a well-known compound in the food industry. Surprisingly, the melanogenic activity of HMF (**1**) was discovered here for the first time. Both HMF and its synthetic analog (**16**) promote the differentiation and migration of melanoblasts in vitro. Typically, stimulator (**1**) upregulated MMP2 expression, which promoted the migration of melanoblasts in vitro.

## 1. Introduction

Vitiligo is an acquired depigmentation disease of the skin [1,2]. Histologically, loss of epidermal melanocytes results in white patches in the skin. The cause is still not clear, although multiple pathogeneses such as autoimmune, neurological, self-destruction, stress, and viral hypotheses are being considered [3,4,5,6]. Patients with vitiligo account for approximately 0.05–1% of the world’s population. Patients often experience difficulties in interpersonal or social relationships due to their appearance, and desperately need an effective therapy to cure vitiligo.

Treatment for vitiligo requires halting of melanocyte destruction and repigmentation of the affected skin. Various methods have been developed to treat vitiligo, including topical or oral steroids [7], immune suppressants [8], phototherapy [9,10], and laser therapy [11]. However, the remaining problems with repigmentation therapies are that repigmentation takes a long time and is usually incomplete. Thus, a more effective medicine is necessary to repigment the lesions. In vitiligo, epidermal melanocytes are lost, but melanoblasts of the outer root sheath of the hair follicles are not affected. Melanoblasts are non-pigmented precursors of melanocytes. They lack tyrosinase and do not stain DOPA or produce melanin. However, upon activation, melanoblasts migrate into the epidermis and differentiate into melanocytes. They then produce melanin pigments. During phototherapy, the repigmentation spots begin from the follicle opening and slowly spread out. Thus, an effective therapeutic agent could be proposed that promotes migration and differentiation of inactive melanoblasts from the outer root sheath of hair follicles to near the epidermis [12,13]. Therefore, melanoblast activation is an ideal target for vitiligo [14,15].

**Figure 1:**
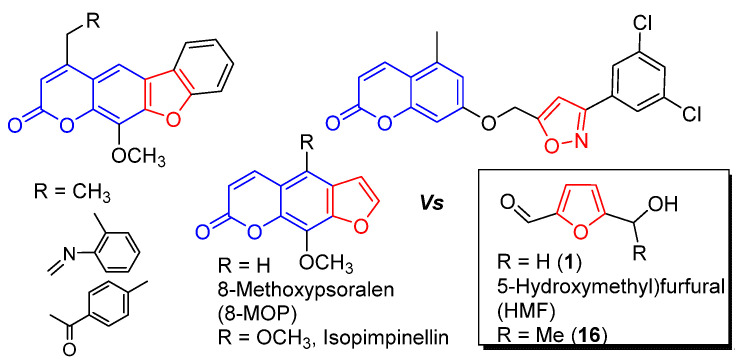
Coumarin (in blue) vs. furan scaffolders for melanogenesis stimulators.

There have been many potential molecules (melanocyte stimulators) that stimulate melanogenesis, mostly at the melanocyte level [16]. However, none of the melanoblast stimulators as single molecules are known except 8-methoxypsoralen (8-MOP) of furocoumarins [17]. Thus far, PUVA therapy (8-MOP plus ultraviolet (UV)A radiation) is still effectively used, but it is being replaced by UVB or laser therapies due to the synergic side effects of 8-MOP (carcinogenesis) [18,19,20]. In addition, the central role of furocoumarins is elusive due to their diverse therapeutic effects [21] and complex nature of pathogenesis. Here, we report a new melanoblast stimulator scaffolder (isolated and characterized from *Dimocarpus longan* and its synthetic analogs) as a furan derivative and a potential substitute for 8-MOP. This new melanoblast activation scaffolder can guide us to view previously reported coumarin derivatives (melanocyte stimulators) [22,23,24] as furan and its analogs having different coumarins (Figure 1). We also showed in vitro that furan derivatives (**1** and **16**) can induce the secretion of matrix metallopeptidase (MMP)-2, which helps in melanoblast migration.

## 2. Results and Discussion

### 2.1. Isolation of HMF

To isolate an active compound of *Dimocarpus longan,* its dried fruit was extracted with methanol at 22 ± 3 °C (rt) to obtain a crude extract, which was suspended in water and hexane. The separated water layers were successively partitioned using CHCl_3_ and EtOAc. Each fraction, including the hexane layer, was evaluated for its ability to induce melanoblast migration, while the extract with CHCl_3_ displayed promising activity (2.1-fold increase in cell migration (%) at 50 μg/mL). The CHCl_3_ extract was further purified by column chromatography on silica gel using a gradient mobile phase (MeOH/CHCl_3_), thereby obtaining three fractions. Among them, the first fraction was further purified by preparative HPLC, which showed the highest migration activity (Figure S1 in ESI). To identify the active chemical structure of the migration activity in *D. longan,* typical characterization techniques, including NMR spectroscopy and high-resolution mass analysis, were conducted. Surprisingly, a few carbon signals were observed in the ^13^C-NMR spectrum (Figure S4 in ESI) and an ion peak was found at *m*/*z* 126.0319 [M]^+^ in the high-resolution mass spectrum (Figure S9 in ESI). These results indicated that the molecule is very small. The coupling constant of the two signals (*J* = 3.5 Hz at 7.45 and 6.56 ppm) in the aromatic region corresponds to a five-membered aromatic ring, while one sharp singlet proton at 9.51 ppm appears to be similar to the proton of the aldehyde group (Figure S3 in ESI). Another set of protons has strong cross-peaks in the COSY NMR spectrum and one of the two peaks at 5.51 ppm is likely attached to a heteroatom because it does not have a cross-peak in HSQC (Figures S5 and S6 in ESI). This structural information enabled the identification of 5-(hydroxymethyl)furfural (HMF). The stacked ^1^H and ^13^C-NMR spectra of the isolated and purchased materials matched exactly (Figures S7 and S8 in ESI). The efficacy profiles of the products isolated from the extract were also similar to those of HMF. Thus, we concluded that the melanogenesis activity of the dried fruit of *D. longan* is responsible for HMF. HMF is a well-known compound in the food industry and is isolated from various plants such as *Schisandra*, *Cornus officinalis*, and *Laurencia undulata* [25]. It is often formed in processed foods such as coffee and dried plum. The toxicity of HMF is not severe, as it is a part of our daily diet, unless it is overconsumed. It has also been reported that there is no adverse effect in the range of 80–100 mg/Kg body weight per day in various animal experiments [26]. Thus, it is a great surprise to us that the melanogenesis activity of HMF was observed for the first time. This finding could be significant from a food and medicinal perspective. 

### 2.2. SAR Study

To support the therapeutic effect of HMF on vitiligo and its preventive effect on gray hair, systematic derivatives were conducted by replacing the two substituents at the α-position of the furan ring in Table 1. The detailed synthetic schemes are in Schemes S1 and S2 (ESI). As stated earlier, melanoblast activation is an ideal target for therapy for vitiligo. Thus, the melanin content of melanoblasts can indicate their differentiation into melanocytes with treatment. Furthermore, measuring the melanin content and migration ability of melanoblasts can be an indicator of the efficacy of the stimulator. In addition, a lower chemical toxicity is necessary to ensure the safety of the stimulator. All these indices were scrutinized through the SAR study. First, the efficacy profile of HMF (**1**) appeared promising as shown in Table 1. The efficacy of the 2, 5-substituted furans was also assessed at a concentration of 10 µM. In the first round, while R_1_ was fixed as the aldehyde group, R_2_ was screened. Replaced ether and amide groups had no effect on melanin content, while cell migration increased slightly, and there was no cytotoxicity (**2**–**6**) as compared to the negative control (DMSO). The ester group increased both melanin content and migration rate (%) compared to the negative control. Surprisingly, the aldehyde group on R_2_ (furan-2,5-dicarbaldehyde (**10**)) decreased both the melanin content and cell migration. Interestingly, the cell viability assay of **10** revealed that it was the most toxic compound in Table 1. The best compound of the first round was the methyl ester (**9**), although both its activities were slightly lower than those of HMF (**1**). A larger ester (**7** and **8**) led to a decrease in both activities compared with compound (**9**). These results indicated that CH_2_OH was the best substitute for R_2_ (**1**). Therefore, CH_2_OH was fixed for R_1_, whereas the other functional groups were examined for R_2_ in the second round. When carboxylic acid derivatives were introduced for R_2_, smaller substituents were better than larger substituents (**13** >**12** > **11**) in terms of melanin content and cell migration. This trend was also observed for the three esters (**7**–**9**). In addition, a one-carbon elongated analog (**15**) of 2,5-furandimethanol (**14**) has an activity profile similar to those of acid derivatives (**11**–**13**) and slightly improved cytotoxicity. It was apparently better than **14**. After the second round, the activities of HMF (**1**) were still the best, and we thought that further derivatives could be limited because smaller derivatives were always better than larger ones. However, one exception was found in the case of **14** vs. **15**. These observations led to the design of compound **16** (one carbon elongation from HMF (**1**)) in the third round, which showed comparable therapeutic indices to HMF. Furthermore, the acetylated compound exhibited a lower activity than compound **16**. Two-carbon elongated compounds (**17**) from HMF decreased the activity compared to one-carbon elongated compounds (**16**). The activities of the acylated compound (**19**) from **17** remained almost the same.

### 2.3. Biological Studies

**Figure 2:**
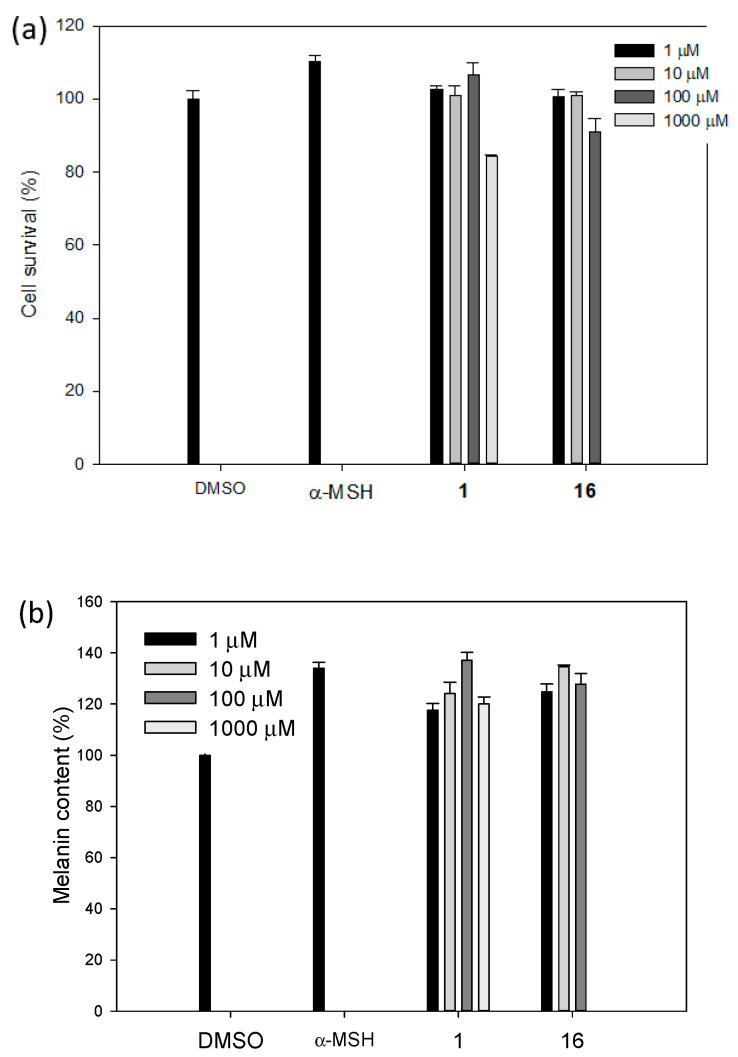

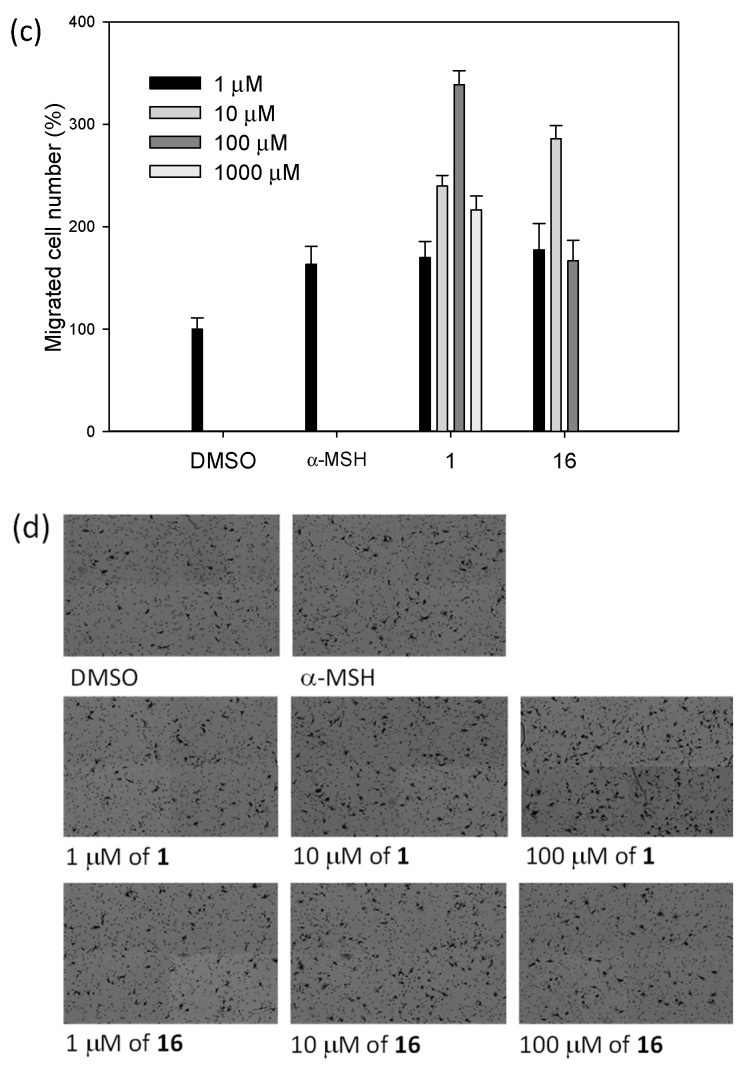
A graph showing the results of evaluating (**a**) cell survival, (**b**) the intracellular melanin content, (**c**) the melanoblast migration at various concentrations of **1** and **16**, (**d**) a set of photographs (magnified 40×) taken after migration assay at various concentrations of **1** and **16**.

Two potential stimulators (**1** and **16**) were selected as representatives from the extract of *D. longan* and the synthetic stimulator, respectively. The cytotoxicity of both compounds at various concentrations was assessed using a typical MTT assay with Melb-a cells (melanoblasts). Stimulator (**1**) showed significant cytotoxicity at 1000 µM, while stimulator (**16**) did at 100 μM in Melb-a (Figure 2a). This observed cytotoxicity should affect the dose dependence of the two therapeutic indices. Thus, the dose dependence of stimulators (**1** and **16**) for the indices was observed in the different concentration ranges (Figure 2). As shown in Figure 2b, stimulator (**1**) displayed dose dependence for the melanin content at a concentration range of 1–100 µM. On the other hand, stimulator (**16**) was dose dependent at a concentration range of 1–10 µM. These trends were also observed in cell migration assays of the two compounds. Compared to the results of the melanin content assay, the dose dependence of cell migration upon treatment with stimulators **1** and **16** was steeper (Figure 2c). Typically, cell migration (%) induced by stimulator **16** was significantly better than that of stimulator **1** below 10 μM. For example, the cell migration rate with stimulator **16** was increased by approximately 2.8 times at a concentration of 10 μM as compared to the negative control, while the migration was increased by about 2.4 times in the case of stimulator **1** under the same conditions. These trends in the cell migration assay were also found with a stimulator (**1** or **16**) at various concentrations. Images were magnified 40 times under a microscope for Figure 2d.

**Figure 3:**
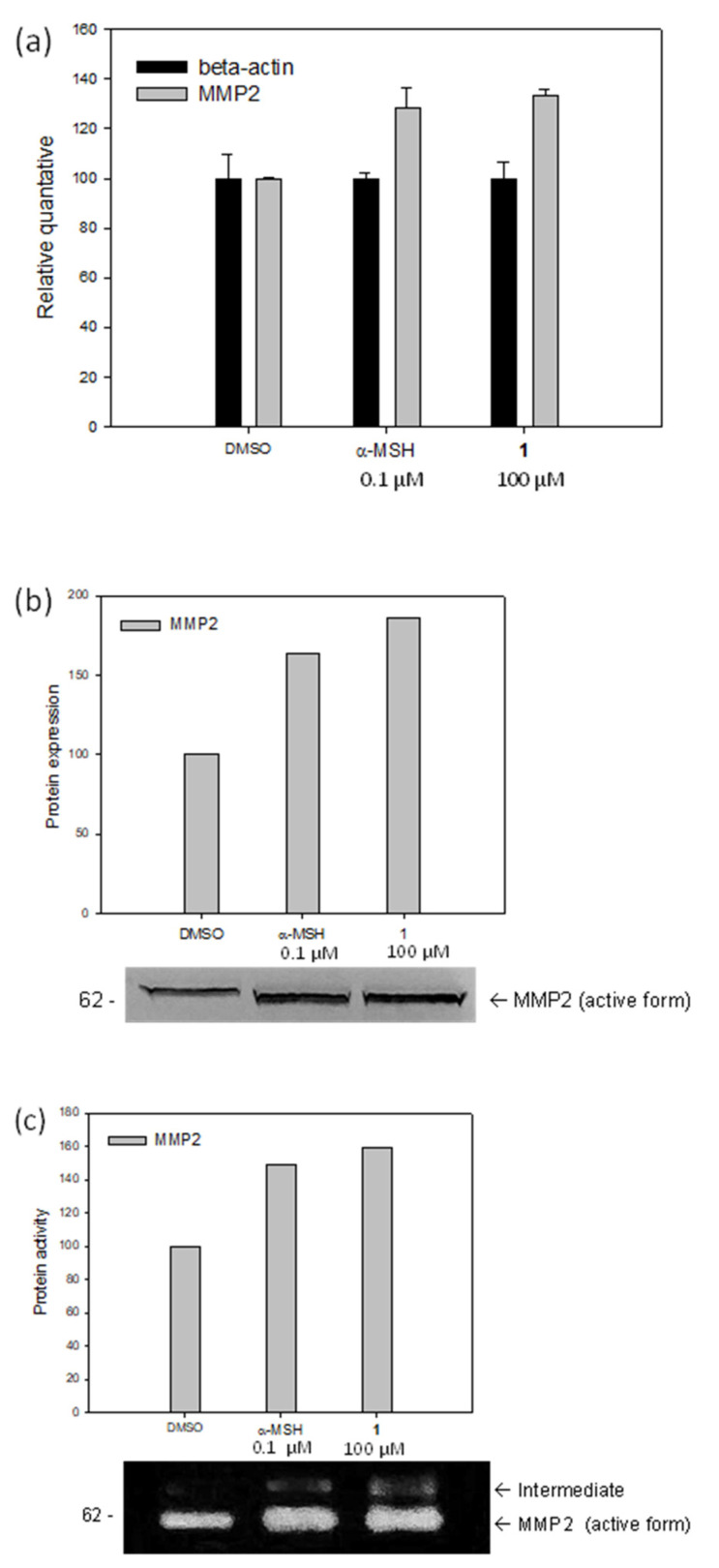
(**a**) mRNA expression of MMP2, (**b**) MMP2 expression, (**c**) gelatinase activity of MMP2 in melanoblasts treated with DMSO, α-MSH, or **1**.

The observed steep dose dependence of cell migration rate may be related to metalloproteinases (MMPs) that play an important role in cleaving components of the extracellular matrix. Previous studies have reported that MMP2 is upregulated when 8-MOP or a-MSH were used in melanoblasts (Melb-a) [17]. Specifically, MMP2, MMP9, and MT1-MMP transcripts are expressed, but MMP2 is overexpressed. In the same study, the percentage of cell migration increased with the overexpression of MMP2, whereas cell migration was reduced upon treatment with additional GM6001 (MMP inhibitor). Thus, we speculated that the steep dose dependence of our stimulators for cell migration was highly related to the overexpression of MMP2. This possibility was thoroughly examined with stimulator **1** as a representative furan derivative and compared with α-MSH (positive control). To identify whether MMP2 is responsible for the migration of melanoblasts in vitro, we first quantified their mRNA expression levels in melanoblasts using semiquantitative RT-PCR when treated with the stimulator (**1**). Both stimulator **1** and α-MSH led to a significant increase in MMP2 mRNA levels (34% and 29%, respectively) compared to the negative control (Figure 3a). In addition, the expression of MMP2 was determined by Western blotting (active form at 62 kDa) with a stimulator (**1**, 100 µM) and α-MSH (0.1 µM) (Figure 3b). 

**Figure 4:**
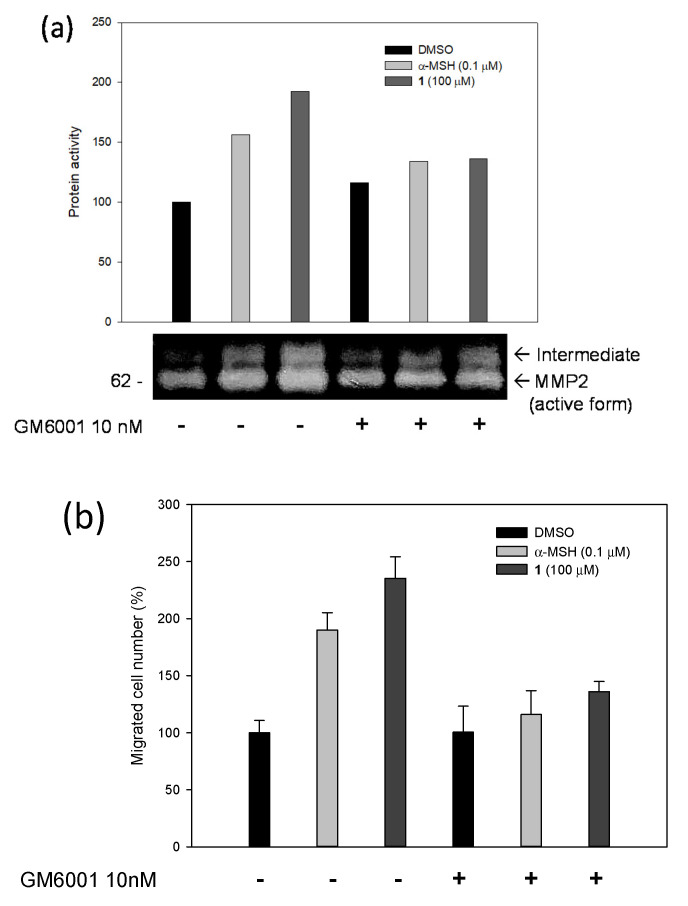
(**a**) MMP2 expression, (**b**) cell migration rate (%) of MMP2 in melanoblasts treated with only **1** (or **1** and GM6001).

MMP2 expression level increased by 86% compared to that in the control when the stimulator (**1**, 100 µM) was used in Melb-a melanoblasts. These results confirm that the expression of MMP2 is induced by this stimulator (**1**). Gelatin zymography was also performed to confirm the proteolytic activity of MMP2 (Figure 3c). When the stimulator (**1**, 100 µM) was used, the protein hydrolysis activity of MMP2 was 60% higher than that of the negative control. Under our experimental conditions, the major MMP secreted by Melb-a melanoblasts was detected using zymography at 62 kDa. This was consistent with the Western blot results (Figure 3b). In contrast, in the presence of both GM6001 (10 nM) and the stimulator (**1**, 100 µM), the protein activity dropped significantly, but not completely, compared to the same experiments performed without GM6001 (Figure 4a). Thus, MMP2 is expressed by melanoblasts, and this expression can be stimulated by the stimulator (**1**, 100 µM), with α-MSH (0.1 µM) as a positive control. To determine whether inhibition of MMP2 affects melanoblast migration, a cell migration assay was conducted with or without GM6001 (10 nM) in the presence of either the stimulator (**1**, 100 µM) or α-MSH (0.1 µM) as a positive control (Figure 4b). Melanoblast migration was enhanced by the stimulator (**1**) and suppressed by GM6001 (1.7 times). These results support that MMP2 is upregulated by the stimulator (**1**) and is crucial for the migration of melanoblasts. It should also be noted that the migratory activity of the stimulator (**1**) is at least 50% higher than that of 8-MOP in Melb-a melanoblasts, compared to the relative value of 8-MOP to α-MSH in the literature [17].

## 3. Materials and Methods

### 3.1. Isolation of Stimulator (***1***)

Isolation procedures of the stimulator (**1**) are summarized in Figure S1 (ESI). It was fully characterized by standard methods, including ^1^H NMR, ^13^C NMR, 2D NMR, and high-resolution mass spectroscopy in the ESI. 

### 3.2. Materials and Synthesis

Reagents were purchased at the highest commercial quality and used without further purification, unless otherwise stated. The yields of the synthesized compounds were measured after chromatographic purification.

The syntheses of all the new compounds are summarized in Schemes S1 and S2 (ESI). All new compounds are characterized by standard methods, including ^1^H NMR, ^13^C NMR, and high-resolution mass spectroscopy. The ^1^H and ^13^C NMR spectra are included in the ESI. 

### 3.3. Plant Material

The dried fruits of *Dimocarpus longan* (Vietnam) were purchased from Kyungdong herbal market (Seoul, Korea) in June 2019 and identified by one of the authors (E.K.K.). A voucher specimen (IUI-2019-06-01) was deposited at the Herbarium of Inha University (IUI), Inha University, Republic of Korea.

### 3.4. Biological Studies

Biological experimental details are available in the ESI (cell culture, MTT assay, cell migration assay, cell differentiation assay, RT-PCR, Western blot analysis, and gelatin zymography).

## 4. Conclusions

We report a new melanoblast stimulator (**1**) scaffolder isolated from *Dimocarpus longan*, and SAR studies of its synthetic analogous support that a new furan scaffolder possibly induces melanogenesis in vitiligo and gray hair because both medical conditions lack melanocytes rather than melanoblasts. Isolated 5-(hydroxymethyl)furfural (HMF, **1**) is a well-known compound in the food industry. Thus, it is surprising that such a small molecule, HMF (**1**), induces melanogenesis in melanoblasts. This is the first report regarding the melanogenic activity of HMF (**1**). Typically, HMF and its synthetic analog (**16**) promote the differentiation and migration of melanoblasts in vitro. Furthermore, the migration rates of the two compounds were at least 50% higher than that of the positive control (8-MOP). Both compounds displayed a step-dose dependence on cell migration rate. Stimulator **1**, a representative furan derivative, promoted cell migration and upregulated MMP2, which is related to the cleavage of the components of the extracellular matrix. In contrast, the cell migration rate decreased when both the stimulator (**1**) and GM6001 (MMP inhibitor) were used simultaneously. Therefore, the mechanism of stimulator **1** in cell migration is suggested to be related to MMP2 upregulation.

## Figures and Tables

**Table 1:** SAR studies of furan derivatives.

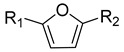					
No.	R_1_	R_2_	Melanin Content ^a^	Cell Migration ^a^	Cell Viability ^a^
**1**	CHO	CH_2_OH	147 (4) ^b^	318 (17) ^b^	108 (4) ^b^
**2**	CHO	CH_2_OCH_2_Ph	82 (4)	114 (19)	104 (2)
**3**	CHO	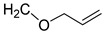	95 (8)	134 (12)	106 (1)
**4**	CHO	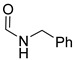	97 (4)	115 (20)	109 (2)
**5**	CHO	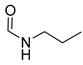	94 (2)	229 (12)	100 (1)
**6**	CHO	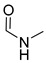	96 (3)	139 (18)	106 (3)
**7**	CHO	CO_2_CH_2_Ph	134 (2)	202 (62)	100 (4)
**8**	CHO	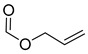	122 (5)	178 (20)	109 (2)
**9**	CHO	CO_2_CH_3_	136 (1)	296 (19)	110 (3)
**10**	CHO	CHO	95 (6)	117 (22)	23 (6)
**11**	CH_2_OH	CO_2_CH_2_CH_3_	104 (5)	111 (14)	71 (4)
**12**	CH_2_OH	CO_2_CH_3_	145 (6)	161 (29)	80 (1)
**13**	CH_2_OH	CO_2_H	158 (1)	264 (8)	93 (1)
**14**	CH_2_OH	CH_2_OH	139 (2)	173 (30)	86 (2)
**15**	CH_2_OH	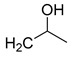	139 (20)	214 (14)	97 (1)
**16**	CHO	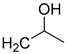	160 (1)	356 (25)	97 (1)
**17**	CHO	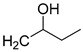	112 (2)	261 (17)	98 (1)
**18**	CHO	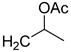	116 (3)	230 (25)	100 (1)
**19**	CHO	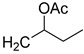	115 (3)	260 (17)	101 (2)

^a^ The values (%) were obtained by comparing the observed effects of negative control (DMSO) for each therapeutic index. ^b^ The numbers in parentheses are measurement errors (±). α-MSH (0.1 μM) was used as a positive control. All assays were conducted in Melb-a (melanoblast) and treated with a 10 μM concentration of each.

## Data Availability

The data presented in this study are available in supplementary material.

## Supplementary Materials

The following supporting information can be downloaded at: https://www.mdpi.com/article/10.3390/molecules27072135/s1, Synthetic details, biological experimental, characterization data of stimulator (**1**), and ^1^H and ^13^C NMR spectra for all new compounds are available online.

## Sample Availability

Samples of the compounds are not available from the authors.
