# Supplementary material for: Isolation of a Melanoblast Stimulator from Dimocarpus longan, Its Structural Modification, and Structure–Activity Relationships for Vitiligo
Source: Molecules. 2022 Mar 25;27(7):2135. doi: 10.3390/molecules27072135 (PMC9000856; doi:10.3390/molecules27072135)
Supplement: Supplementary file 1 [file molecules-27-02135-s001.zip › molecules-1643444-supplementary.pdf]

Supporting Information

Isolation of a Melanoblast Stimulator from *Dimocarpus longan*, its Structural Modification, and Structure−Activity Relationships for Vitiligo

Jae-Won Song,[†] Sunju Choi,[‡] Gayeong Kim,[‡] Hyang Bok Lee,[‡] P. Sankara Rao,[†] Jeonghyun Shin,[¶] Eun Ki Kim,[‡] and Dong-Gyu Cho*,[†]

dgcho@inha.ac.kr

[†]Department of Chemistry and Chemical Engineering, Inha University, Functional Molecule Synthesis Laboratory, Incheon 22212, Republic of Korea

[‡]Department of Biological Engineering, Inha University, Incheon 22212, Republic of Korea

[¶]Department of Dermatology, School of Medicine, Inha University, Incheon 22212, Republic of Korea

Contents

Synthetic Details **S2**

Isolation and Characterization of melanoblast stimulator (1) from *Dimocarpus longan* **S5**

Biological Experimental **S12**

NMR Spectra of furan derivatives **S14**

Synthetic Details

General

Reagents were purchased at the highest commercial quality and used without further purification, unless otherwise stated. Yields of synthesized compounds were measured after chromatographic purification. ^{1}H, ^{13}C, and 2D-NMR spectra were measured at 25 ºC using 400 MHz spectrometers. HRMS were recorded by EI methods using a magnetic sector-electric sector double focusing analyzer.

Scheme S1. Synthetic scheme of furan derivatives from HMF

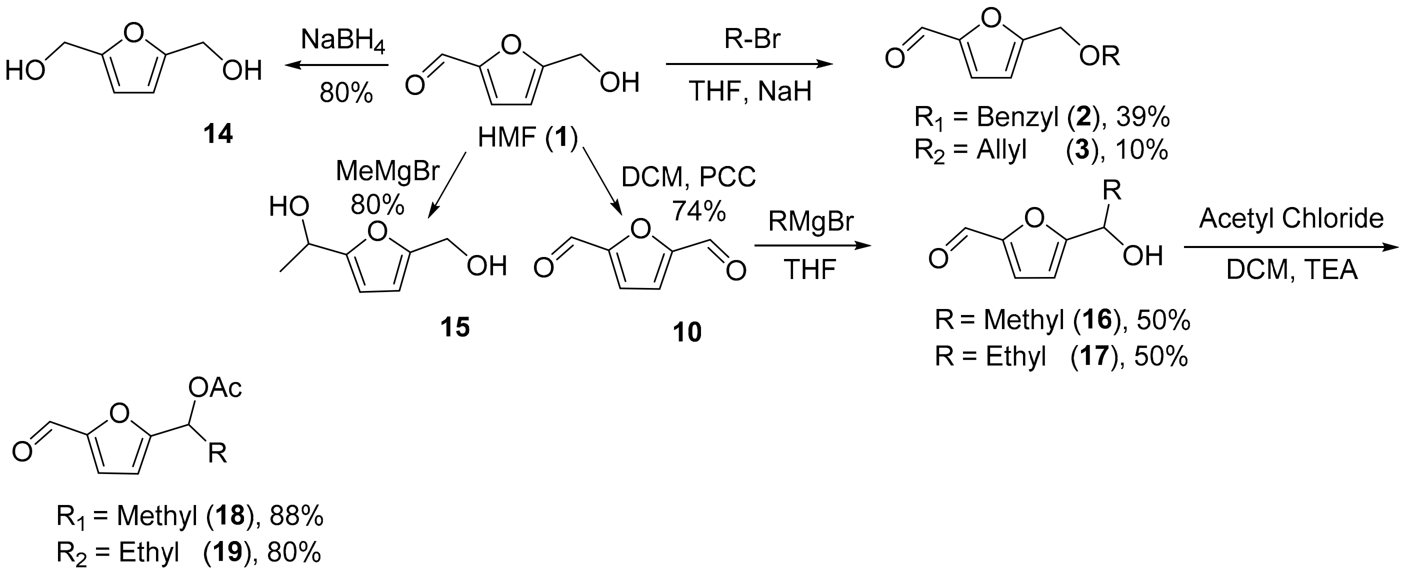

Scheme S2. Synthetic scheme of furan derivatives from 2-Formyl-5-furancarboxylic acid

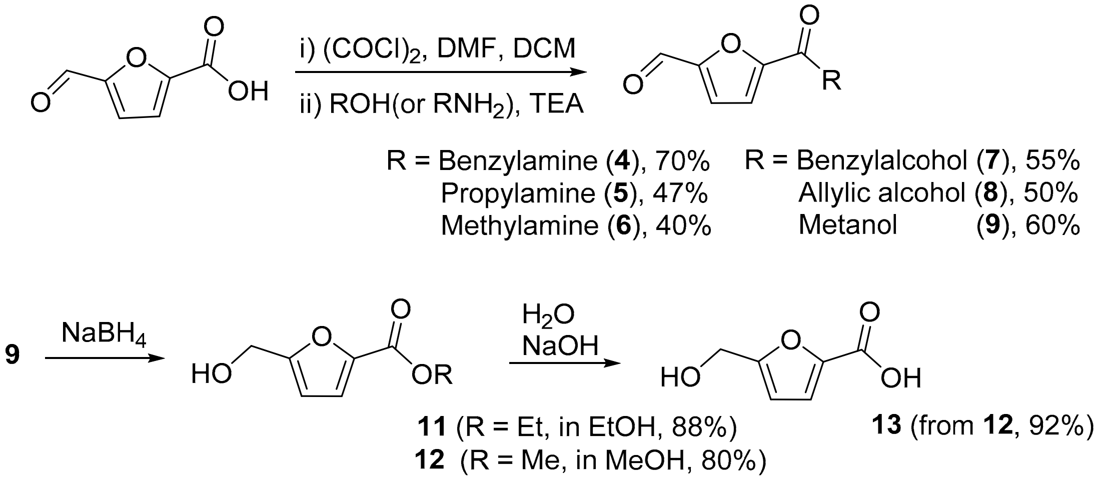

Synthetic procedure

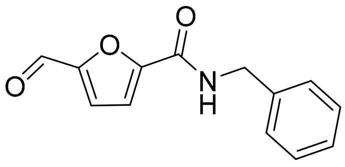

N-benzyl-5-formylfuran-2-carboxamide (4)

5-formylfuran-2-carboxylic acid (100 mg, 0.71 mmol) was dissolved in DCM (2 ml) in inert condition. After oxalyl chloride (0.092 ml, 1.07 mmol) was added dropwise, few drops of DMF was added until bubbling occur. The mixture was stirred for 1 hour at room temperature. The solution was cooled to 0 °C. Benzyl amine (0.23 ml, 2.14 mmol) and TEA (0.3 ml, 2.14 mmol) was added to the solution. The solution was stirred for 2 hours at 0 °C and evaporated to dryness. The reaction was diluted in DCM and organic layer was washed with water and 1 M of aqueous HCl solution. The organic layer was dried over Na_2SO_4 and evaporated to dryness. The residue was purified over silica gel (Ethyl Acetate:Hexane = 1:1) to afford the compound (115 mg, 70%). ^{1}H NMR (400 MHz, $CDCl_3$) δ 9.64 (s, 1H), 7.31 (s, 7H), 7.21 (d, *J* = 3.7 Hz, 1H), 6.92 (s, 1H), 4.59 (d, J = 5.9 Hz, 2H); ^{13}C NMR (100 MHz, $CDCl_3$) δ 178.00, 157.25, 152.31, 151.30, 137.30, 128.92, 128.15, 127.96, 122.30, 115.76, 43.57; HRMS–EI: m/z $[M]^+$ calcd for $C_{13}H_{11}NO_3$: 229.0739; found: 229.0741.

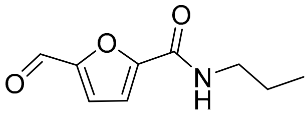

5-formyl-N-propylfuran-2-carboxamide (5)

5-formylfuran-2-carboxylic acid (100 mg, 0.71 mmol) was dissolved in DCM (2 ml) in inert condition. Oxalyl chloride (0.092 ml, 1.07 mmol) was added dropwise and DMF was added few drops until bubbling occur. The mixture was stirred for 1 hour at room temperature. The solution was cooled to 0 °C. Propyl amine (0.18 ml, 2.14 mmol) and TEA (0.3 ml, 2.14 mmol) was added in solution. The mixture was stirred for 1 hour 0 °C and evaporated to dryness. The reaction was diluted in DCM and organic layer was washed with water and 1 M of aqueous HCl solution. The organic layer was dried over Na_2SO_4 and evaporated to dryness. The residue was purified over silica gel (Ethyl Acetate:Hexane = 1:1) to afford the compound (60 mg, 47%). ^{1}H NMR (400 MHz, $CDCl_3$) δ 9.67 (s, 1H), 7.27 (d, *J* = 3.7 Hz, 1H), 7.21 (d, *J* = 3.7 Hz, 1H), 6.76 (s, 1H), 3.39 (dt, *J* = 7.2, 6.3 Hz, 1H), 1.62 (tq, *J* = 7.2 Hz, 2H), 0.96 (t, *J* = 7.2 Hz, 2H); ^{13}C NMR (100 MHz, $CDCl_3$) δ 178.08, 157.44, 152.18, 151.67, 122.62, 115.35, 41.25, 30.98, 22.84, 11.43; HRMS–EI: m/z $[M]^+$ calcd for $C_9H_{11}NO_3$: 181.0739; found: 181.0741.

.

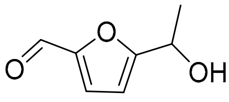

5-(1-hydroxyethyl)furan-2-carbaldehyde (16)

Furan-2,5-dicarbaldehyde (98 mg, 0.79 mmol) was dissolved in THF (4 ml) in inert condition. The mixture was cooled to -20 °C and 3 M of CH_3MgBr (0.13 ml, 0.395 mmol in Et_2O) was added dropwise. The reaction was stirred for 1 hour at -20 °C and quenched with saturated NH_4Cl. The reaction was diluted in ethyl acetate and organic layer was washed with water and brine. The organic layer was dried over Na_2SO_4 and evaporated to dryness. The residue was purified over silica gel (Ethyl Acetate:Hexane = 1:1) to afford the compound (55 mg, 50%). ^{1}H NMR (400 MHz, $CDCl_3$) δ 9.57 (s, 1H), 7.19 (d, *J* = 3.6 Hz, 1H), 6.46 (d, *J* = 3.6 Hz, 1H), 4.95 (q, *J* = 6.4 Hz, 1H), 2.61 (very broad, 1H), 1.58 (d, *J* = 6.4 Hz, 3H); ^{13}C NMR (100

MHz, $CDCl_3$) δ 177.72, 164.49, 152.12, 122.81, 108.02, 63.96, 21.56; HRMS–EI: m/z $[M]^+$ calcd for $C_7H_8O_3$: 140.0473; found: 140.0471.

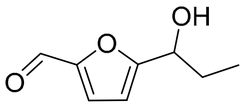

5-(1-hydroxypropyl)furan-2-carbaldehyde (17)

Furan-2,5-dicarbaldehyde (100 mg, 0.81 mmol) was dissolved in THF (1 ml) in inert condition. The mixture was cooled to -20 °C and 1 M of C_2H_5MgBr (0.405 ml, 0.405 mmol in THF) was added dropwise. The reaction was stirred for 1 hour at the same temperature and quenched with saturated NH_4Cl. The reaction was diluted in ethyl acetate and organic layer was washed with water and brine. The organic layer was dried over Na_2SO_4 and evaporated to dryness. The residue was purified over silica gel (Ethyl Acetate:Hexane = 1:1.5) to afford the compound (62 mg, 50%). ^{1}H NMR (400 MHz, $CDCl_3$) δ 9.58 (s, 1H), 7.20 (d, *J* = 3.5 Hz, 1H), 6.47 (d, *J* = 3.5 Hz, 1H), 4.71 (dd, *J* = 7.2, 5.8 Hz, 1H), 2.00 – 1.80 (m, 2H), 0.98 (t, *J* = 7.4 Hz, 3H); ^{13}C NMR (100 MHz, $CDCl_3$) δ 177.60, 163.61, 152.23, 122.56, 108.68, 69.41, 28.90, 9.61; HRMS–EI: m/z $[M]^+$ calcd for $C_8H_{10}O_3$: 154.0630; found: 154.0632.

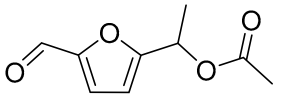

1-(5-formylfuran-2-yl)ethyl acetate (18)

5-(1-hydroxyethyl)furan-2-carbaldehyde (50 mg, 0.36 mmol) was dissolved in DCM (1 ml). TEA (0.05 ml, 0.36 mmol) was added in the solution. The mixture was cooled to 0 °C and acetyl chloride (0.026 ml, 0.36 mmol) was added dropwise. The reaction was allowed to warm to room temperature and stirred for 4 hours. The solution was quenched with 5% aqueous HCl. The organic layer was washed with water and brine and dried over Na_2SO_4 and evaporated to dryness. The residue was purified over silica gel (Ethyl Acetate:Hexane = 1:2) to afford the compound (57 mg, 88%). ^{1}H NMR (400 MHz, $CDCl_3$) δ 9.61 (s, 1H), 7.18 (d, *J* = 3.6 Hz, 1H), 6.49 (d, *J* = 3.6 Hz, 1H), 5.95 (q, *J* = 6.7 Hz, 1H), 2.07 (s, 3H), 1.61 (d, *J* = 6.8 Hz, 4H). ^{13}C NMR (100 MHz, $CDCl_3$) δ 177.86, 169.99, 159.66, 152.47, 121.78, 110.18, 65.08, 21.06, 18.43; HRMS–EI: m/z $[M]^+$ calcd for $C_9H_{10}O_4$: 182.0579; found:182.0576.

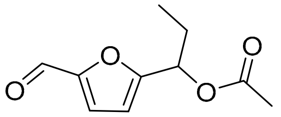

1-(5-formylfuran-2-yl)propyl acetate (19)

5-(1-hydroxypropyl)furan-2-carbaldehyde (11 mg, 0.07 mmol) was dissolved in DCM (0.2 ml). TEA (0.01 ml, 0.08 mmol) was added in the solution. The mixture was cooled to 0 °C and acetyl chloride (0.006 ml, 0.08 mmol) was added dropwise. The reaction was allowed to warm to room temperature and stirred for 4 hours.The solution was quenched with 5% aqueous HCl. The organic layer was washed with water and brine and dried over Na_2SO_4 and evaporated to dryness. The residue was purified over silica gel (Ethyl Acetate:Hexane = 1:2) to afford the compound (11 mg, 80%). ^{1}H NMR (400 MHz, $CDCl_3$) δ 9.62 (s, 1H), 7.19 (d, *J* = 3.6 Hz, 1H), 6.49 (d, *J* = 3.5 Hz, 1H), 5.79 (t, *J* = 7.0 Hz, 1H), 2.10 (s, 3H), 2.06 – 1.96 (m, 2H), 0.93 (t, *J* = 7.4 Hz, 3H); ^{13}C NMR (100 MHz, $CDCl_3$) δ 177.80, 170.15, 159.00, 152.50, 121.69, 110.78, 69.97, 25.98, 20.97, 9.56; HRMS–EI: m/z $[M]^+$ calcd for $C_{10}H_{12}O_4$: 196.0736; found: 196.0733.

Isolation and Characterization of melanoblast stimulator (1) from *Dimocarpus longan*

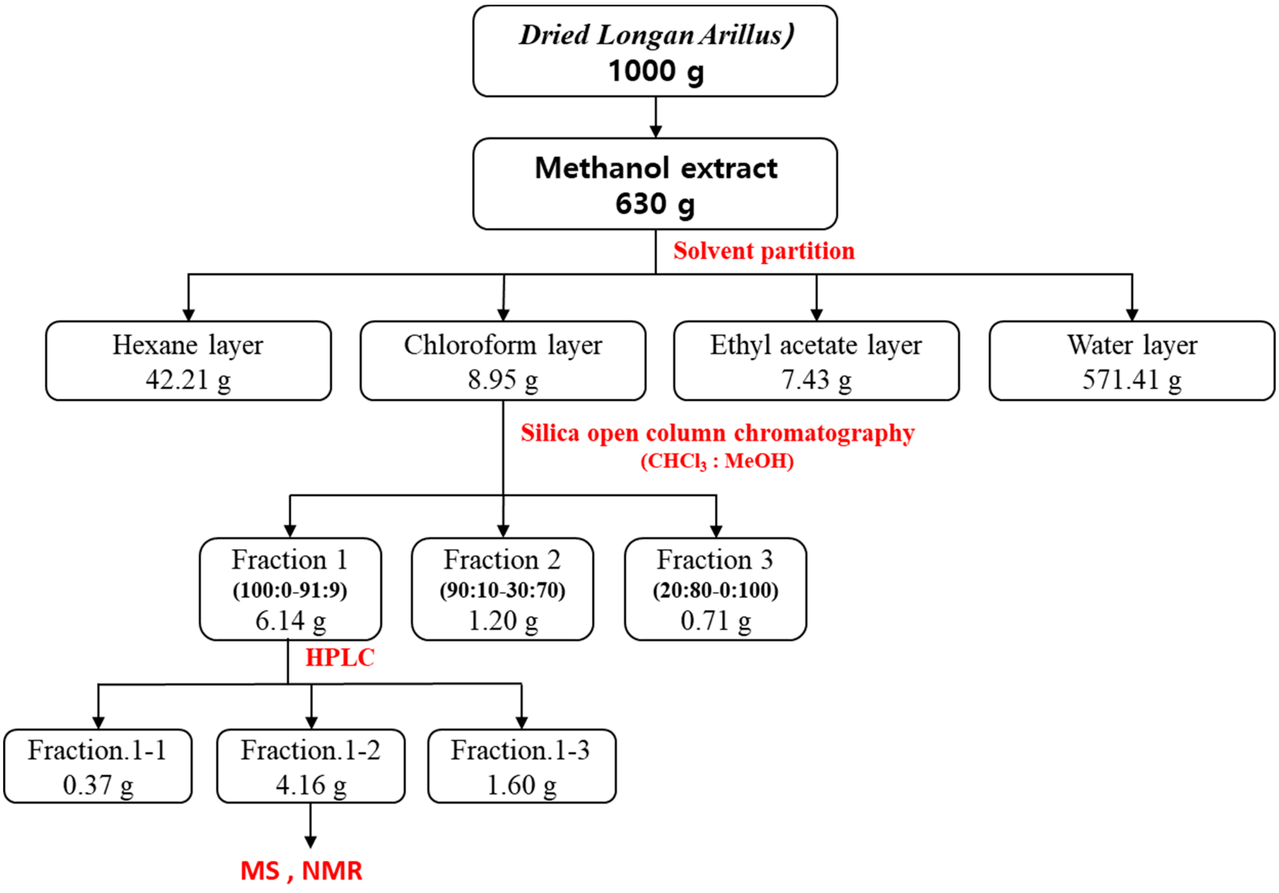

Figure S1. Summaried purification procedures for HMF (**1**)

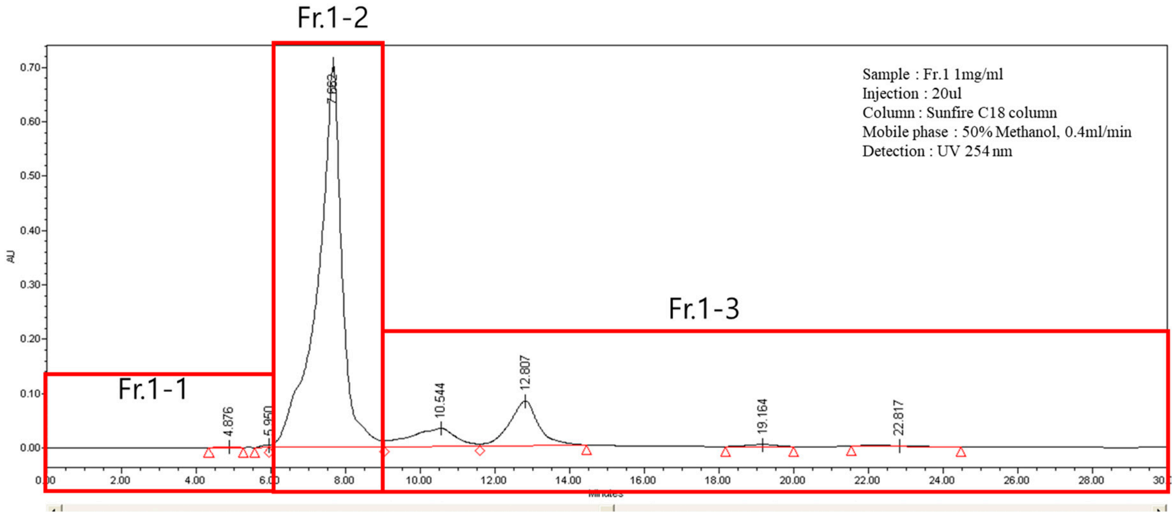

Figure S2. HPLC profile of the mixture after column chromatography (Sunfire C18 column (Waters, 3.5 µm, 4.6 × 150 mm, USA; 50% Methanol containg water (isoratic); 4 ml/min.). The faction of 1-2 (Fr.1-2) contains HMF (**1**).

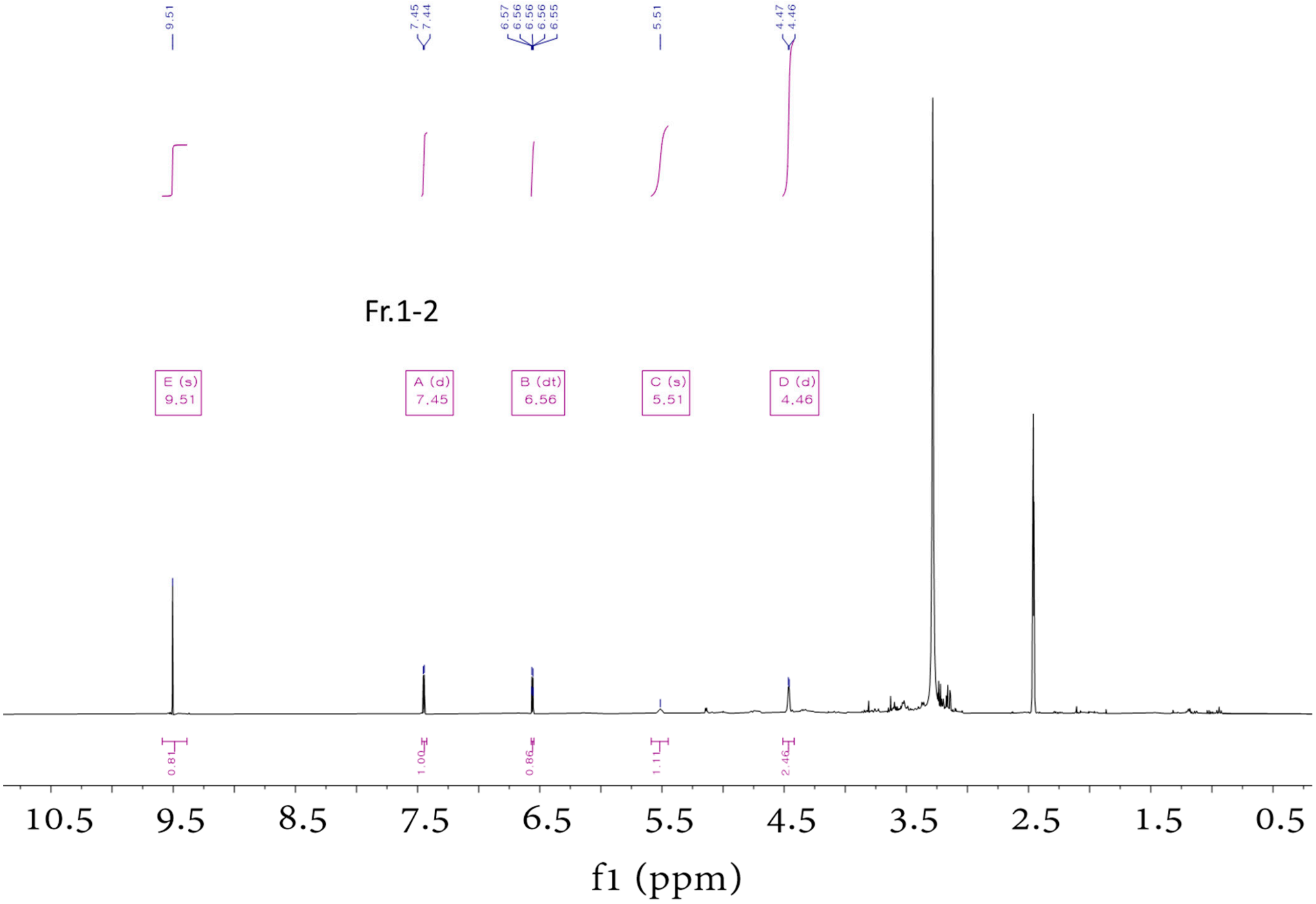

Figure S3. ^{1}H NMR spectrum of **Fr.1-2** in DMSO-d_6 at 25 ºC (400 MHz)

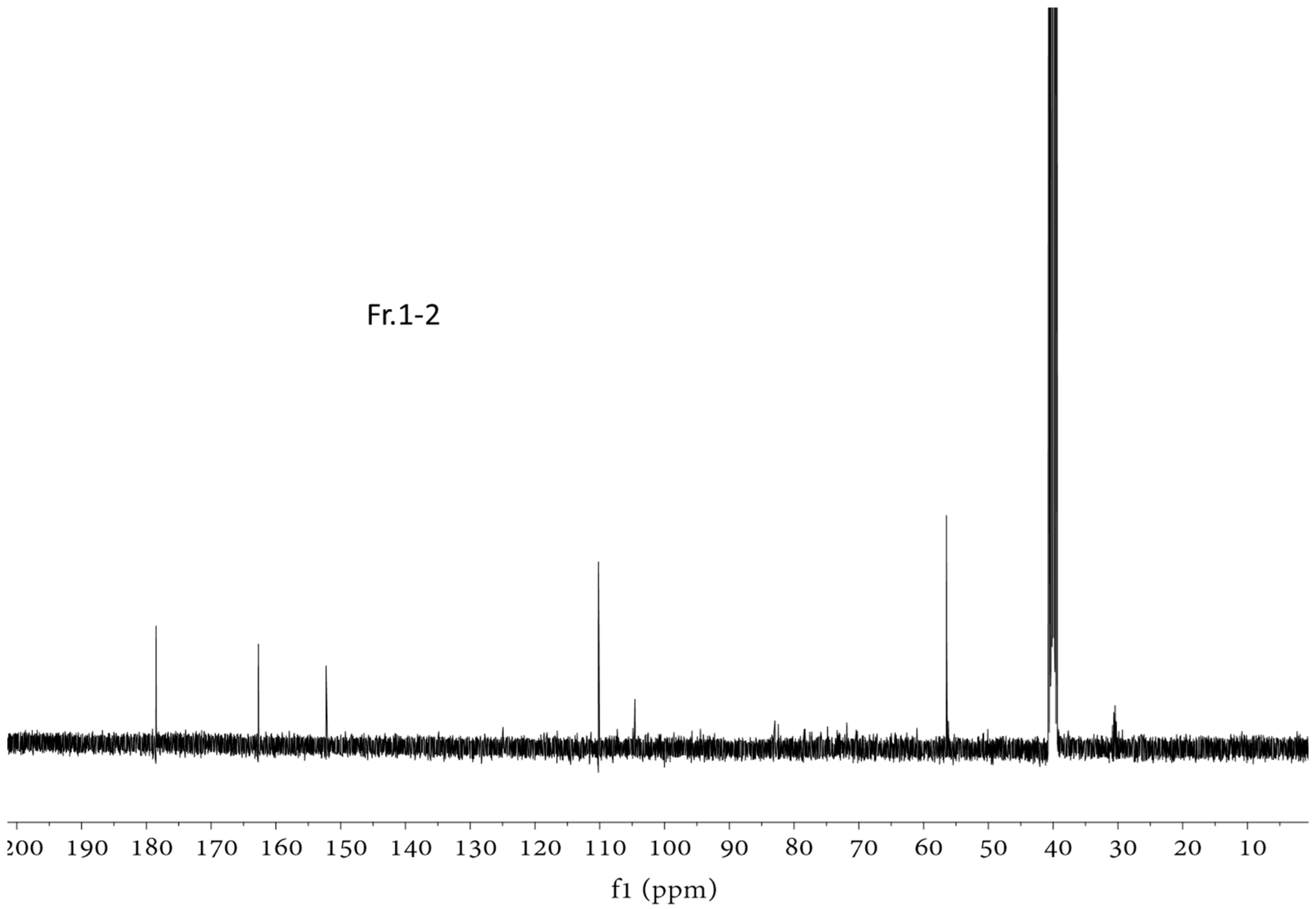

Figure S4. ^{13}C NMR spectrum of **Fr.1-2** in DMSO-d_6 at 25 ºC (100 MHz)

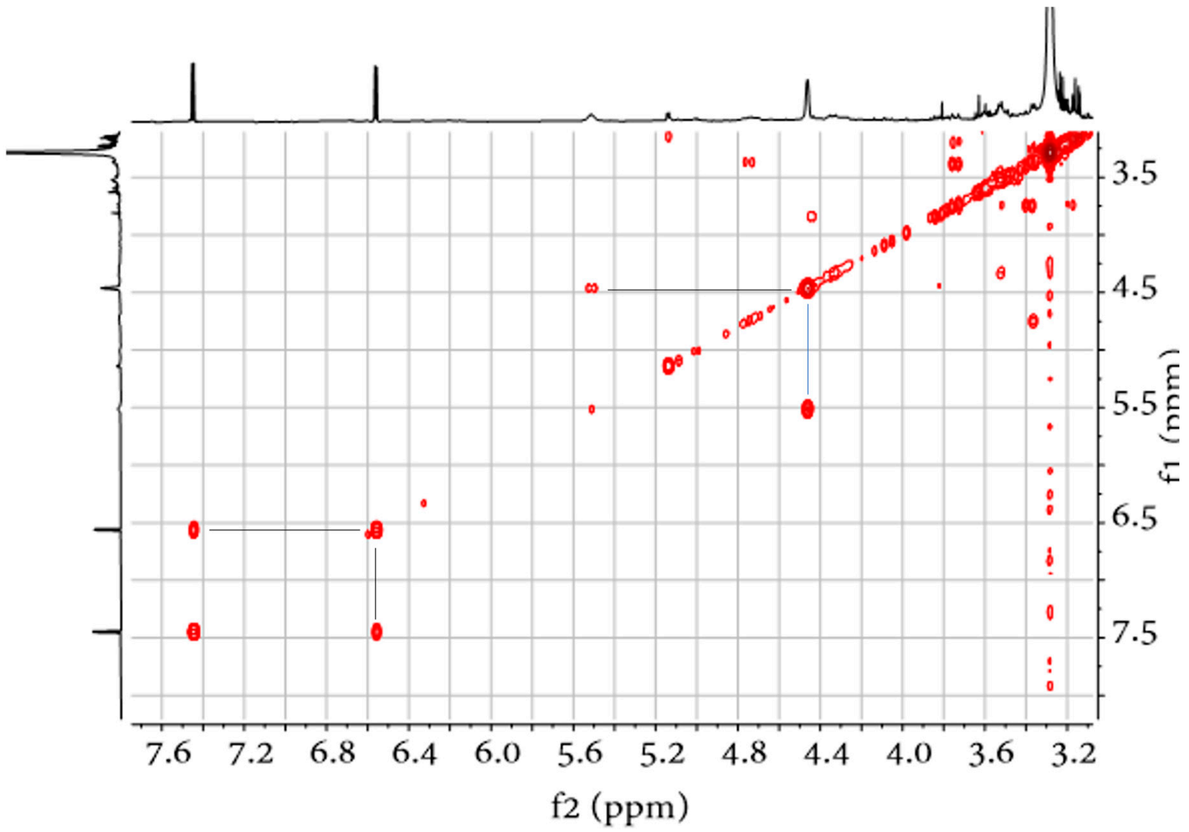

Figure S5. 2D COSY NMR spectrum of **Fr.1-2** in DMSO-d_6 at 25 ºC (400 MHz)

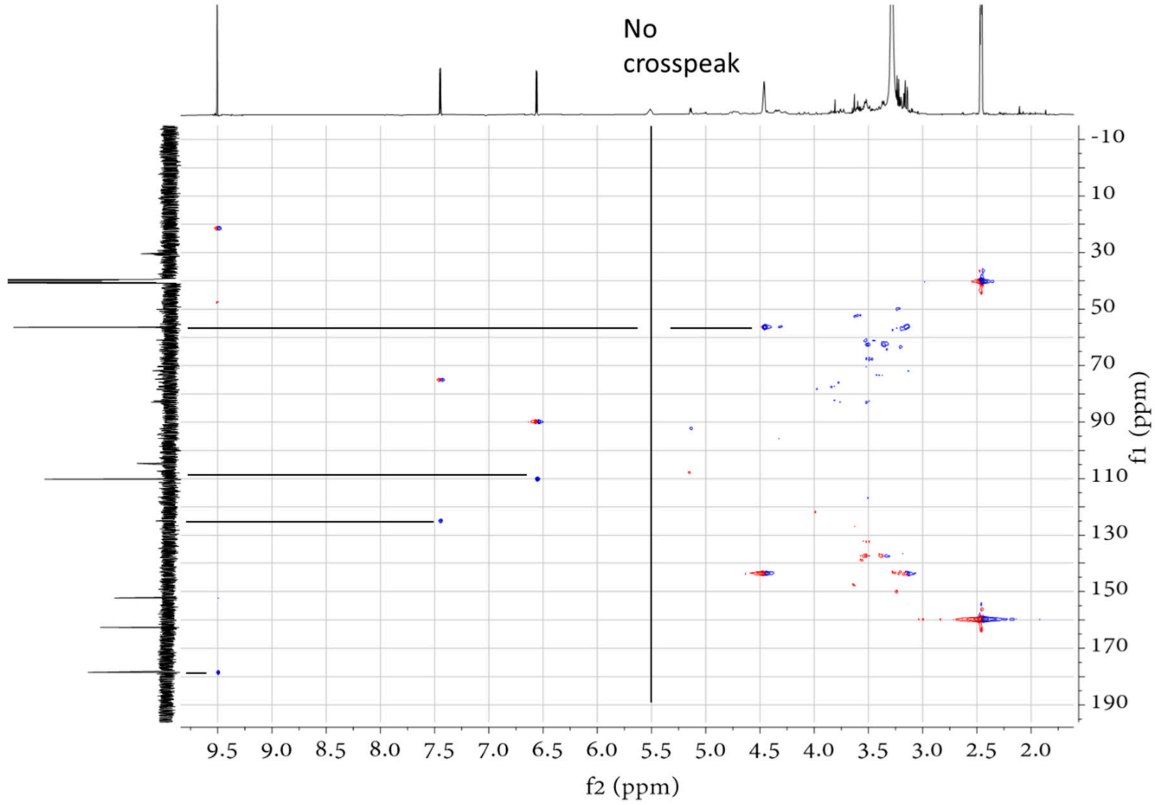

Figure S6. 2D HSQC NMR spectrum of **Fr.1-2** in DMSO-d_6 at 25 ºC (400 MHz)

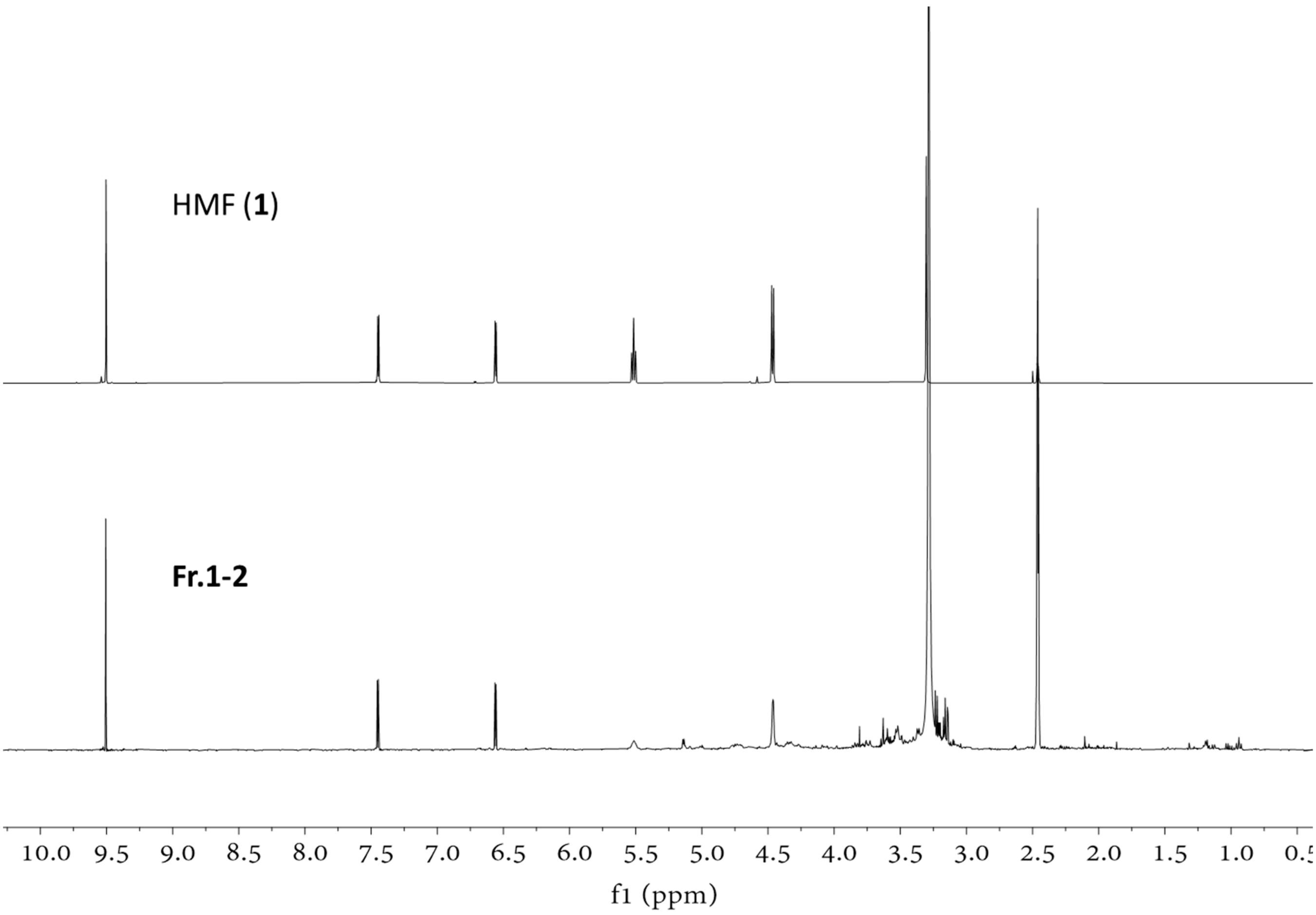

Figure S7. Stacked ^{1}H NMR spectra of **Fr.1-2** and HMF (**1**) in DMSO-d_6 at 25 ºC (400 MHz)

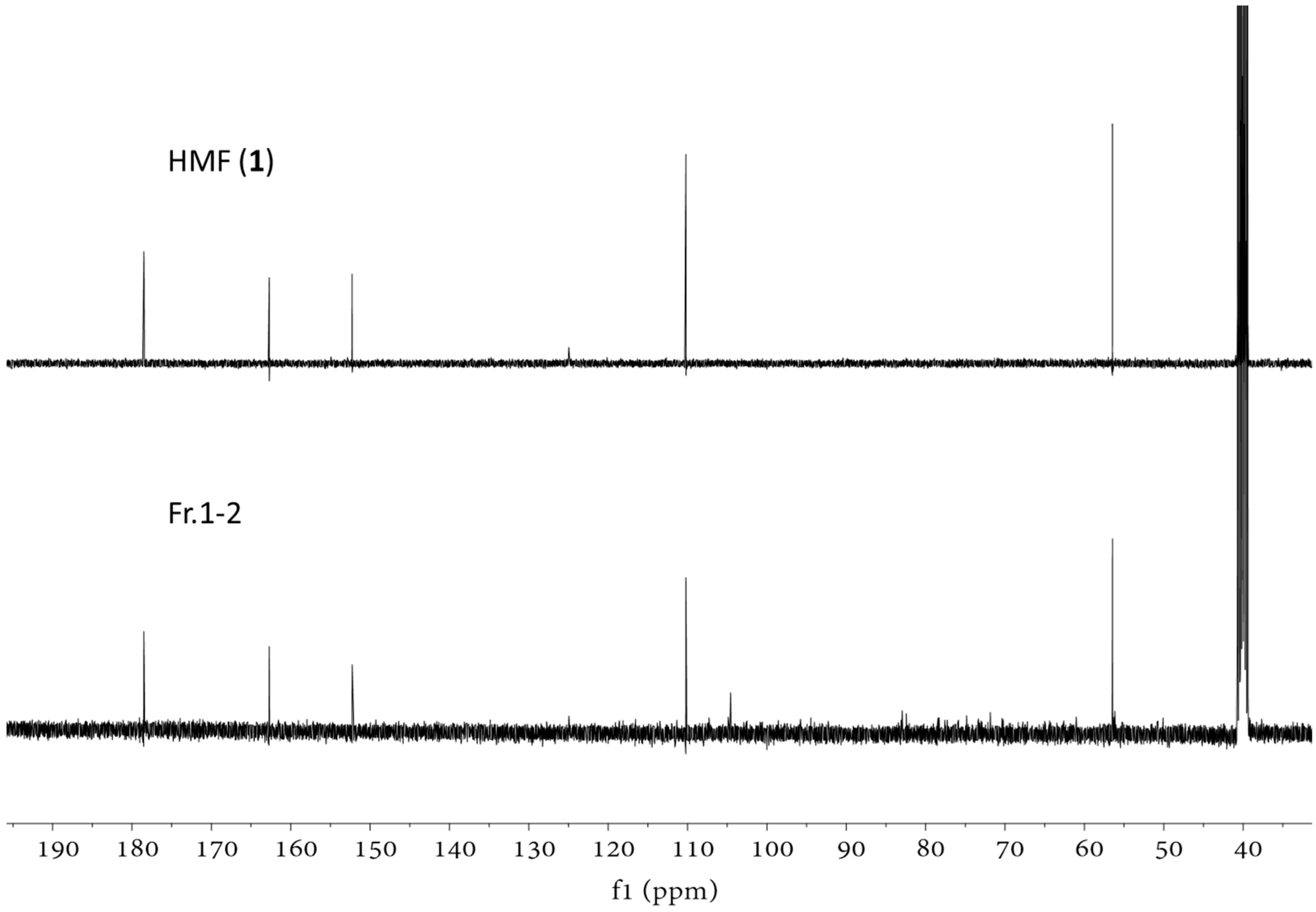

Figure S8. Stacked ^{13}C NMR spectra of **Fr.1-2** and HMF (**1**) in DMSO-d_6 at 25 ºC (100 MHz)

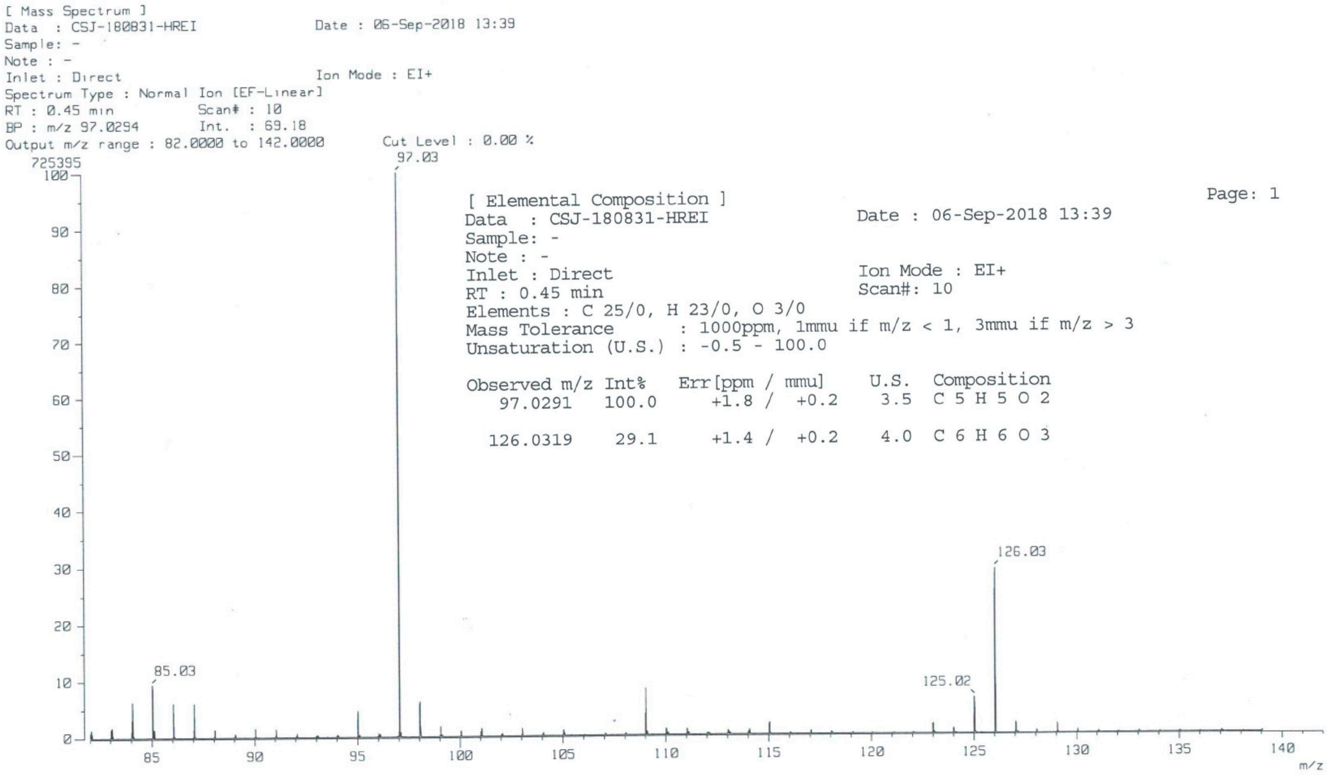

Figure S9. Theoretical and observed HRMS data of **Fr.1-2** (HRMS (EI): m/z $[M]^+$ calcd for $C_6H_6O_3$:126.0317; found: 126.0319)

Biological Experimental

Materials

Phorbol 12,13 dibutyrate (PDBu), dimethyl sulfoxide (DMSO), α-melanocyte-stimulating hormone (α-MSH), gelatin B, 3-(4,5-dimethylthiazole-2-yl-2,5-diphenyl tetrazolium bromide) (MTT), and Trizma® base were purchased from Sigma Chemical Co. (St Louis, MO, USA). Basic fibroblast growth factor (bFGF) and GM6001 (Galardin) were purchased from PeproTech (New Jersey, USA) and Abcam (Cambridge, MA, USA), respectively. HMF was purchased from Tokyo Chemical Industry Co. Ltd. (Tokyo, Japan). RPMI 1640, trypsin-EDTA, fetal bovine serum (FBS), phosphate-buffered saline (PBS), and penicillin/streptomycin were obtained from Invitrogen Co., Ltd. (CA, USA).

Cell culture

Melb-a cells were obtained from the Wellcome Trust Functional Genomics Cell Bank (London, UK). The cells were cultured in a RPMI 1640 medium supplemented with 1% penicillin/streptomycin (100 unit/mL), 5% fetal bovine serum (GibcoBRL, Rockville, MD, USA), PDBu (20 nM), and bFGF (1 ng/mL) at 37 °C in humidified air with a 10% CO_2 atmosphere.

MTT assay

Melb-a cells were cultured at 6 x 10^3 cells in a 12-well plate at 37 °C in a placed in a 10% CO_2 incubator for 24 h, exchanged for fresh complete medium containing the compound (1, 10, 100, and 1000 µM), and cultured under the same conditions for 4 days. As a control group, DMSO and 100 nM of α-MSH (α-Melanocyte-stimulating hormone) were used for the negative and positive controls. After 96 h, 100 µL of MTT (3-(4,5-dimethylthiazol-2-yl)-2,5-diphenyltetrazoliumbromide, Sigma Chemical Co., St Louis, USA) at a concentration of 5 mg/mL was added, and the cultures were incubated for 4 h at 37 °C a 10% CO_2 incubator. The medium was removed, and the remaining formazan crystals in the cells were dissolved in DMSO. The absorbance of each well was measured at 540 nm wavelength using an ELISA microplate reader.

Cell migration assay

The transwell cell culture chamber was used to assess whether cell migration was induced by compound treatment. In the transwell cell culture chambers (Costar 3422; Cambridge, MA, USA), polyvinylpyrrolidone-free polycarbonate filters with an 8.0 um pore size were precoated with 1% gelatin. After hardening the coated cell, 600 µL of serum-free RPMI medium was added to the bottom of the chamber, and 100 µL of melb-a cells (2 × 10^5 cells) was inoculated in the upper chamber, followed by culturing for 24 h. After 24 h, the filter was removed, and the cells were fixed with methanol. After 24 h, the filter was fixed with 70% methanol and stained with hematoxylin and 5% eosin. The cells remaining on the upper surface of the filters were removed by wiping with a cotton swab, and the cells that migrated to the bottom of the filter were counted under a microscope and photographed. To measure the number of cells, the filter was divided into quarters and observed under a microscope at 40 times magnification, and the average value was obtained. The experiment was repeated twice for each condition.

Cell differentiation assay (Melanin contents assay)

Melb-a cells were seeded at 6 x 10^4 cells in 6-well plates. After 24 h incubation at 37 °C, the medium was replaced with a fresh one and the samples were treated at different concentrations and cultured for 48 h. The culture supernatant was then removed and washed with 1 mL PBS (phosphate buffered saline, Invitrogen Co. CA, USA) per well. After detaching the cells with trypsin-EDTA, cells were harvested by centrifugation. Cell pellets were dissolved in 1 N NaOH containing 10% DMSO at 80 °C for 1 h. The amount of melanin was measured at 405 nm using a multi-plate reader and was expressed as a percentage compared to the control.

RT-PCR

Total mRNA was extracted from cultured cells using the RNeasy Total RNA Isolation Kit (QIAGEN, Santa Clarita, CA, USA) according to the manufacturer's instructions. cDNA was synthesized by mixing 1 μg of total RNA, 4 μL of RT/RI enzyme mix, and 1 μg of gDNA remover and reacting with Uvigen at 42 °C for 15 min, 85 °C for 5 s, and 4 °C for 3 min. The synthesized cDNA was amplified using a PikoReal 96 Real-Time PCR system (Thermo Scientific, Madison, WI, USA). Ten microliters of 2X SYBR Green PCR master mix (QIAGEN), 20 nM forward of the corresponding gene, 0.5 μL of reverse primer, 1 μL of template cDNA, and distilled water were mixed to prepare a 20 μL of mixture. Real-time PCR was performed for 40 cycles at 95 °C for 5 min, 95 °C for 30 s, 58 °C for 30 s, and 60 °C for 5 min. Primers used in the experiment were purchased from Bioneer (Daejeon, Korea). For specific MMP-2 amplification, the oligonucleotides 5′-GGCTCTCCAGAACATCATCC-3′ (forward) and 5′-GGGTFTCGCTGTTGAAGTCA-3′ (reverse) were used. For specific human glyceraldehyde-3-phosphate dehydrogenase (GAPDH) cDNA amplification, oligonucleotides 5′-AACGGTCGGGAATACAGCAG-3′ (forward) and 5′-AAACAAGGCTTCATGGGGGC-3′ (reverse) were used. The relative MMP-2 mRNA expression level was calculated by normalizing to GAPDH expression level.

Western blot analysis

Western blot analysis was performed to determine MMP-2 expression levels as previously described. The extracellular secreted MMP-2 protein and cell culture medium were collected and concentrated using Amicon® Ultra-15 Centrifugal Filters Ultracel® 10 K and quantified using the BCA assay. Twenty micrograms of total protein from each cell were electrophoresed in reducing SDS-PAGE and blotted on PVDF membranes (Immobilon-P, Millipore Corp, Bedford, MA, USA) using a transblot system. The membranes were blocked with 5% skim milk in Tris-buffered saline containing 0.1% Tween-20 (TBST, pH 7.6) for 2 h at 22±3 °C and then reacted with primary antibodies (Cell Signaling Technology, Danvers, MA, USA) overnight at 4 °C. After washing three times with TBST, the blots were incubated with HRP-conjugated rabbit anti-goat secondary antibodies (Santa Cruz, Dallas, USA), and reacted HRP signals were detected with the TMB membrane peroxidase substrate (KPL, Gaithersburg, MD, USA) solution. To study MMP-2 inhibition, GM6001 (10 nM) was co-treated with all samples during cell culture, and western blotting was performed in the same manner as described above.

Gelatin zymography

Gelatin zymography was performed to confirm the levels of MMP-2 activity in melanoblasts treated with the compound compared with the untreated DMSO control. MMP-2 protein was obtained in the same way as described above and subjected to SDS-PAGE using 1 mg/mL gelatin (Sigma Aldrich, USA) containing a 10% polyacrylamide gel. After electrophoresis, gels were equilibrated in Tris-HCl buffer containing 2.5 % Triton X-100 for 1 h at 22±3 °C. Subsequently, for gelatinase activity, the gels were incubated in 50 mM Tris-HCl (pH 7.5), 10 mM $CaCl_2$, and 150 mM NaCl buffer at 37 °C for 24 h to induce gelatin hydrolysis by MMP-2 protein. The gel was stained with 0.5% Coomassie Brilliant Blue R 250 (Thermo Scientific) and decolorized after 30 min. The proteolytic activity of MMP 2 was detected as a transparent band in contrast to the color of the blue-stained gel. For the study of MMP 2 inhibition, GM6001 (10 nM) was co-administered to all samples during cell culture and zymography was performed in the same manner as described above.

NMR Spectra of furan derivatives

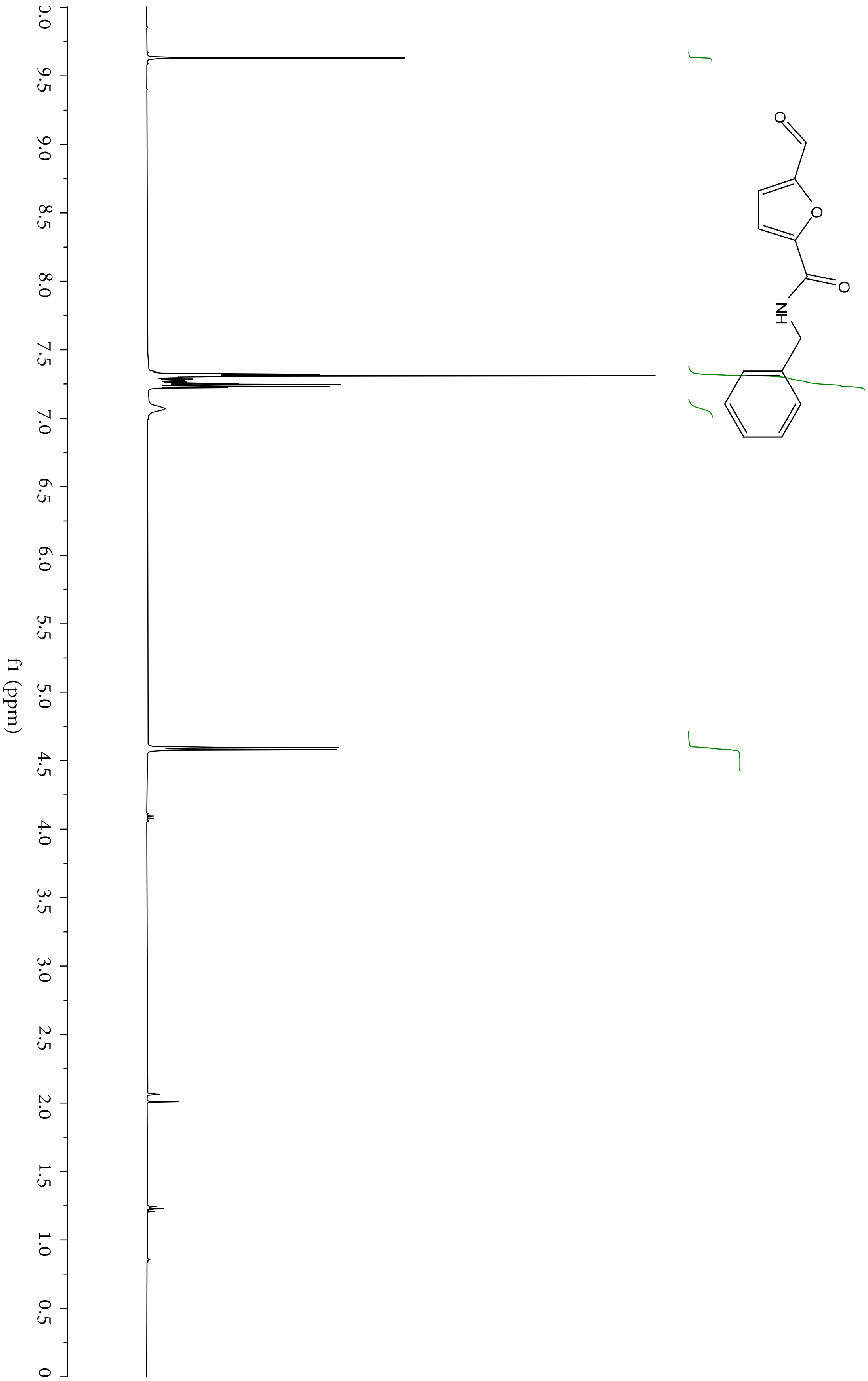

Figure S10. ^{1}H NMR spectrum of **4** recorded in $CDCl_3$ at 25ºC (400 MHz)

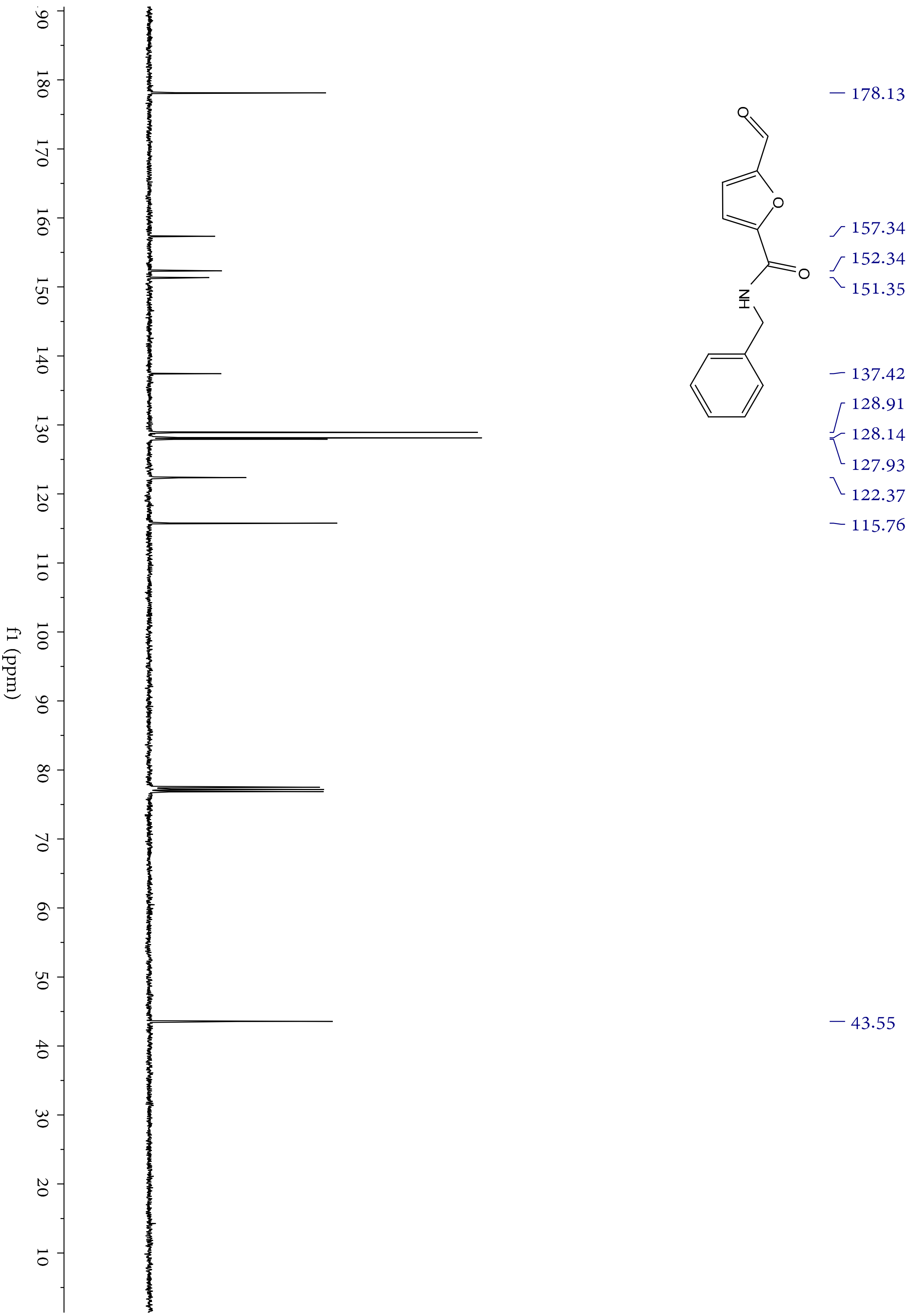

Figure S11. ^{13}C NMR spectrum of **4** recorded in $CDCl_3$ at 25°C (100 MHz)

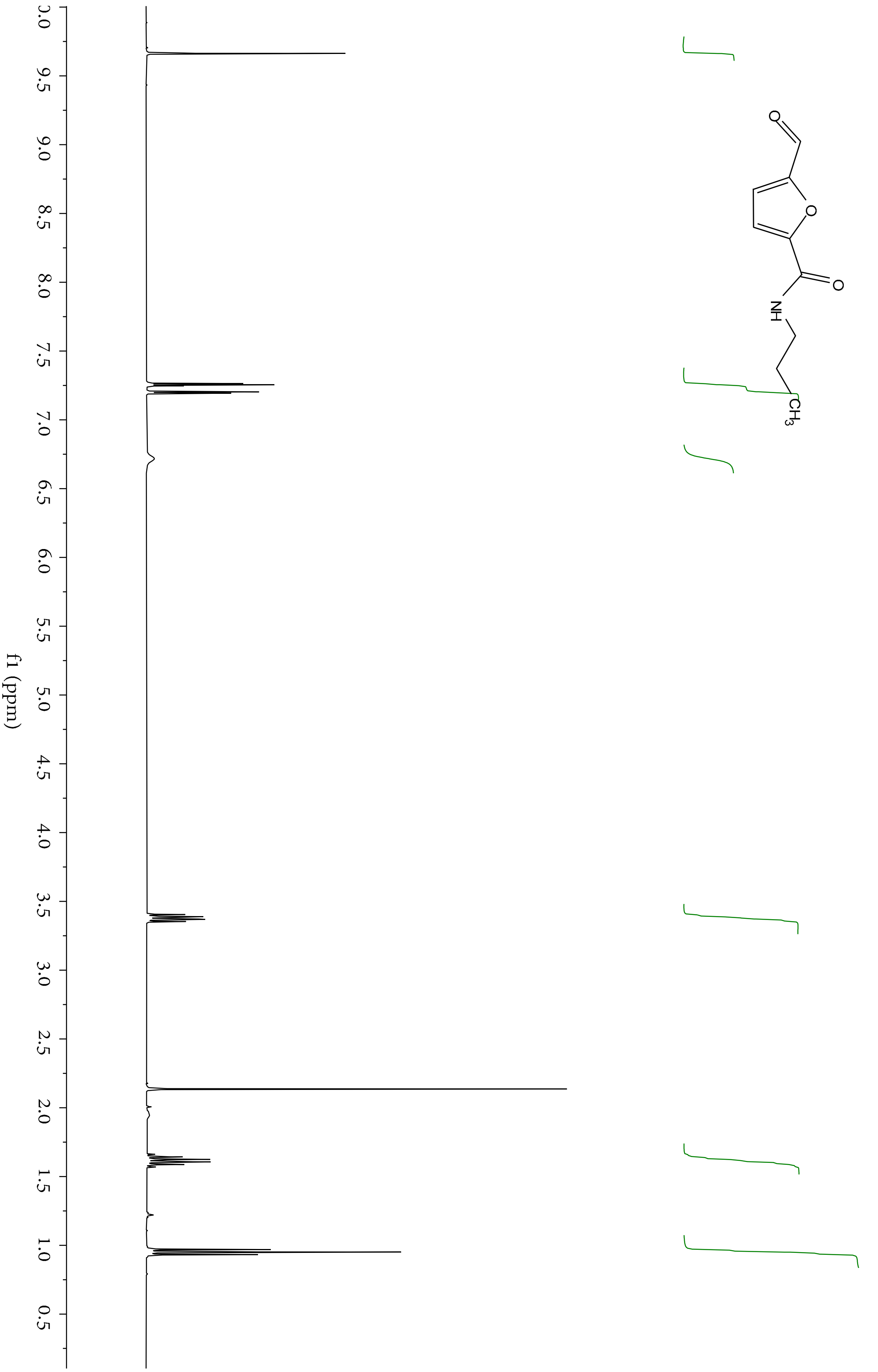

Figure S12. ^{1}H NMR spectrum of **5** recorded in $CDCl_3$ at 25ºC (400 MHz)

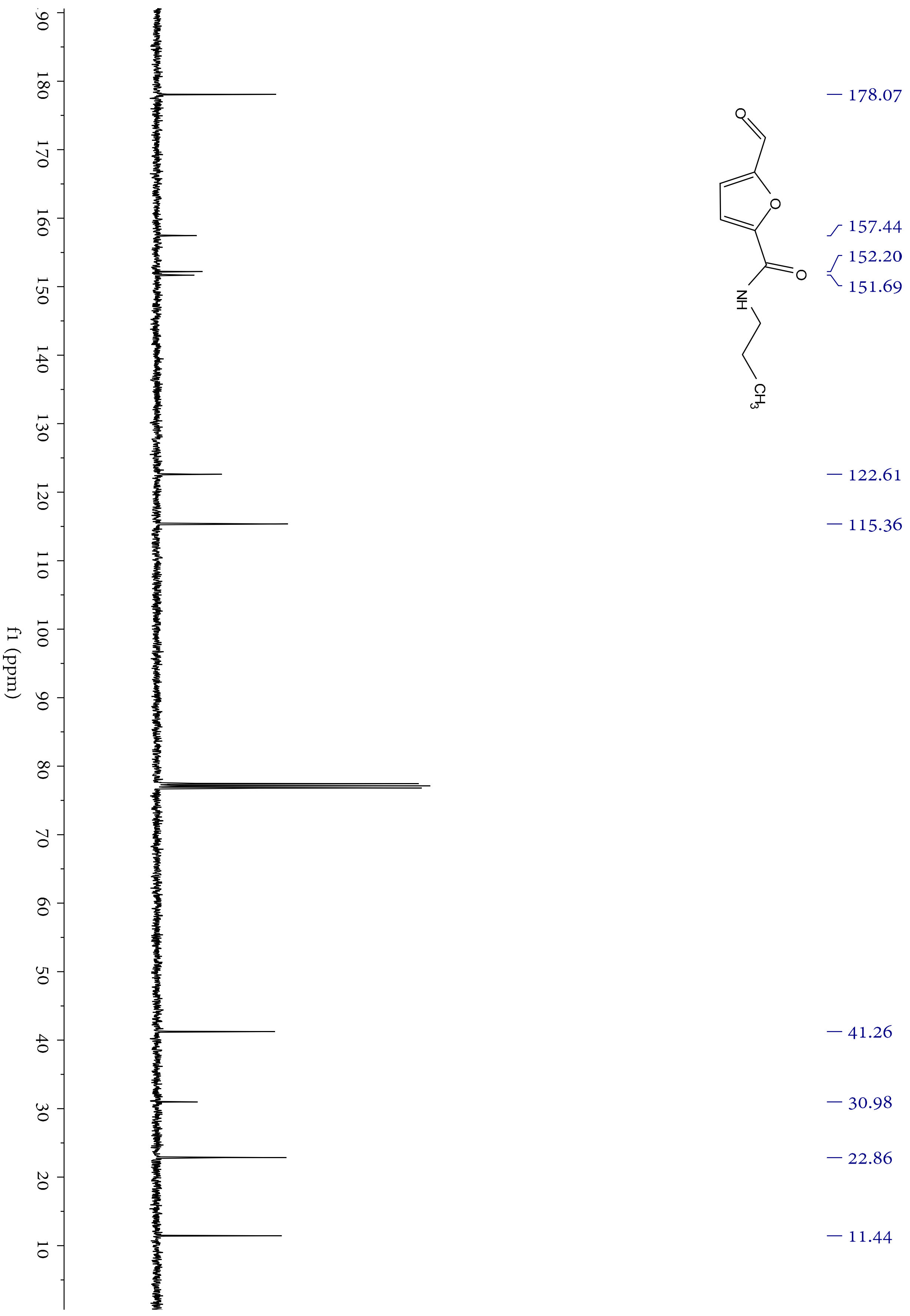

Figure S13. ^{13}C NMR spectrum of **5** recorded in $CDCl_3$ at 25ºC (100 MHz)

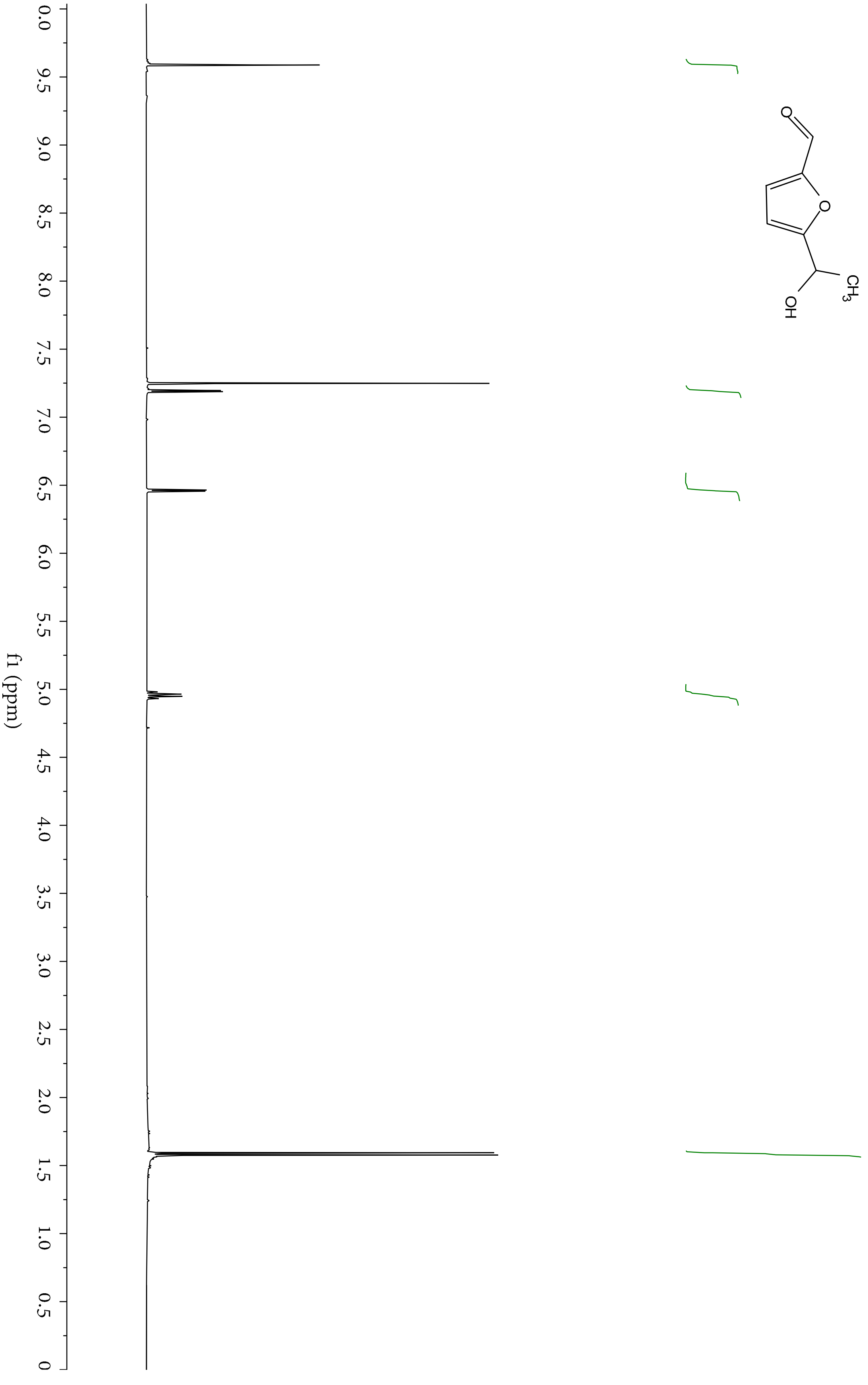

Figure S14. ^{1}H NMR spectrum of **16** recorded in $CDCl_3$ at 25ºC (400 MHz)

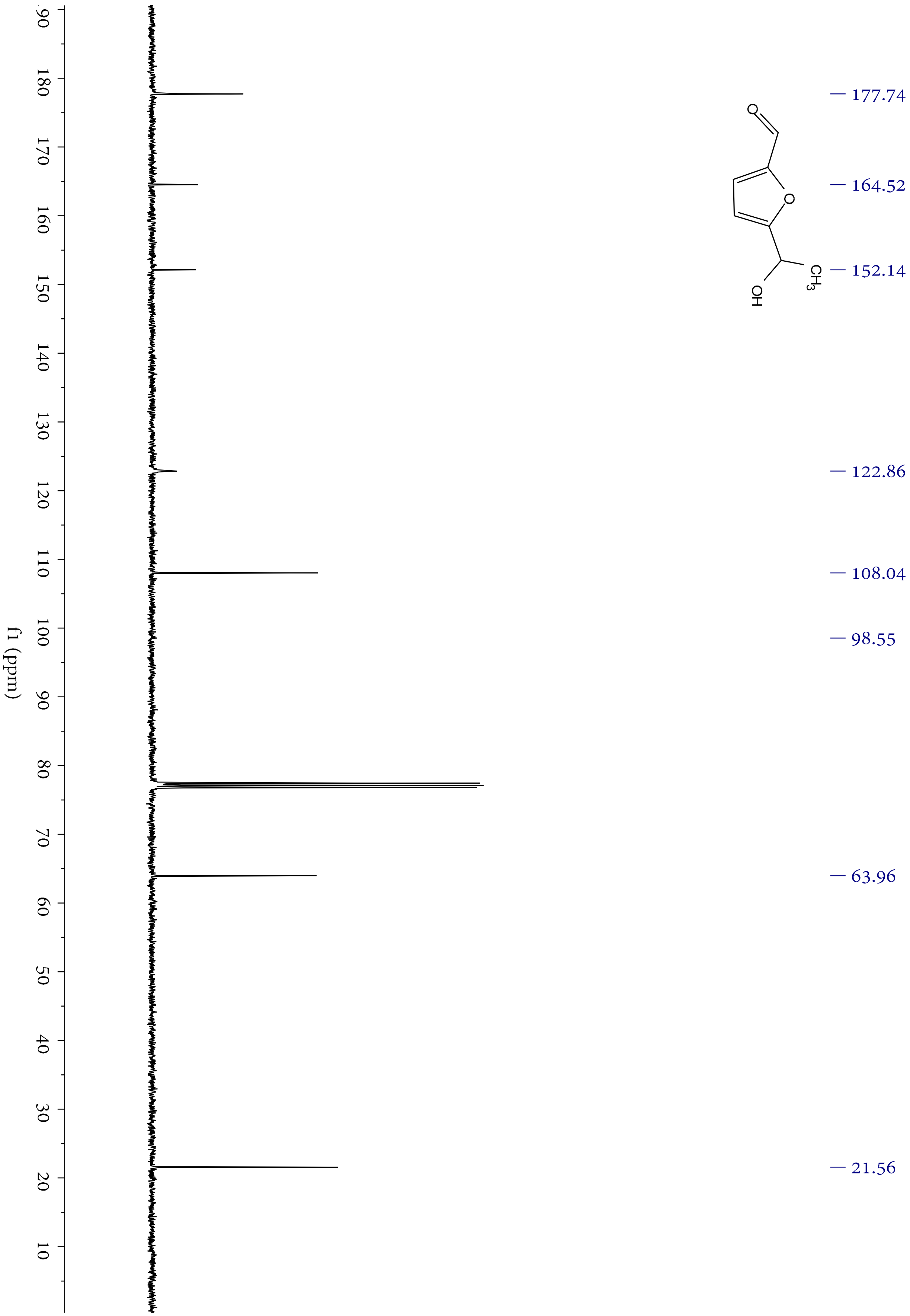

Figure S15. ^{13}C NMR spectrum of **16** recorded in $CDCl_3$ at 25°C (100 MHz)

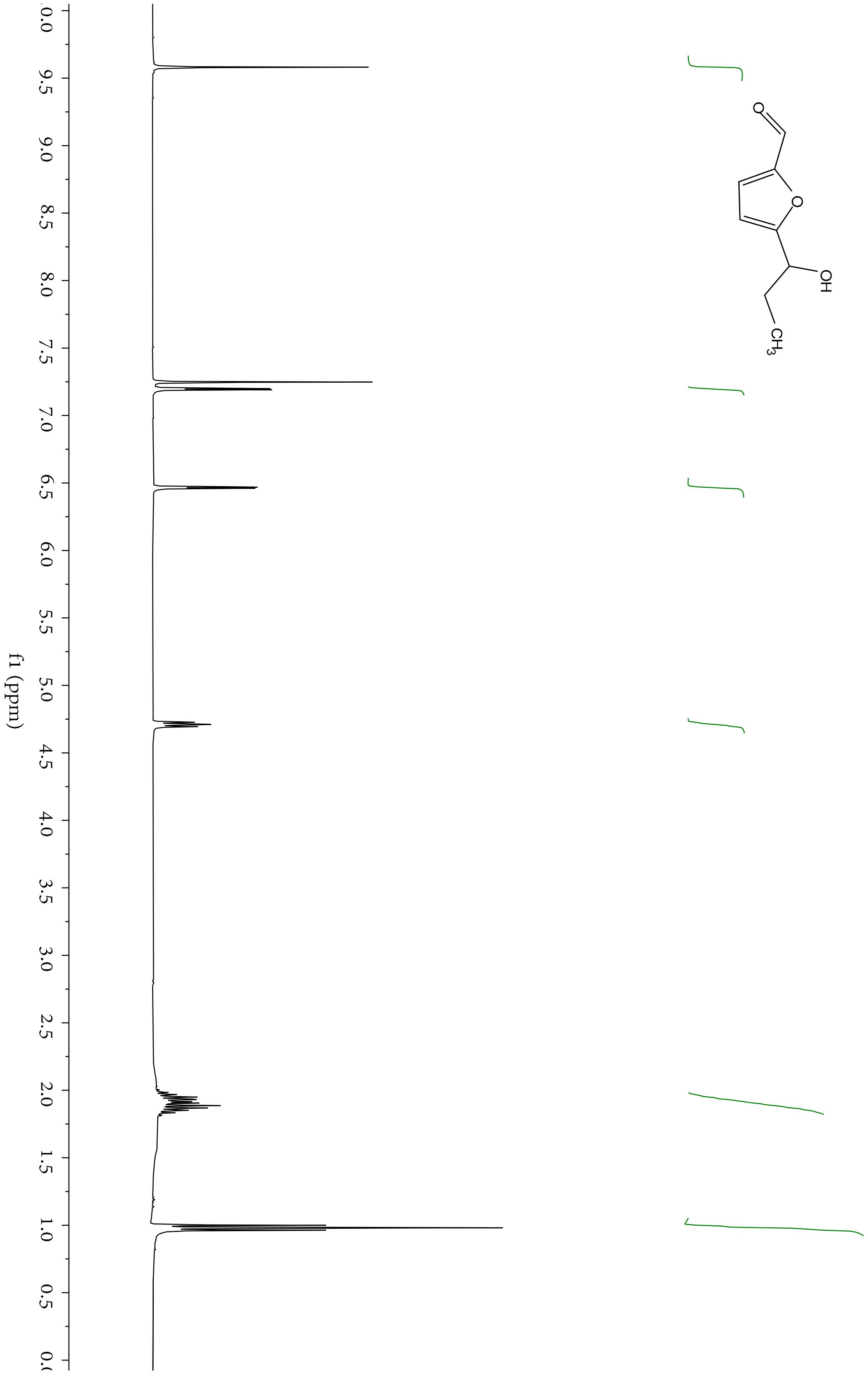

Figure S16. ^{1}H NMR spectrum of **17** recorded in $CDCl_3$ at 25ºC (400 MHz)

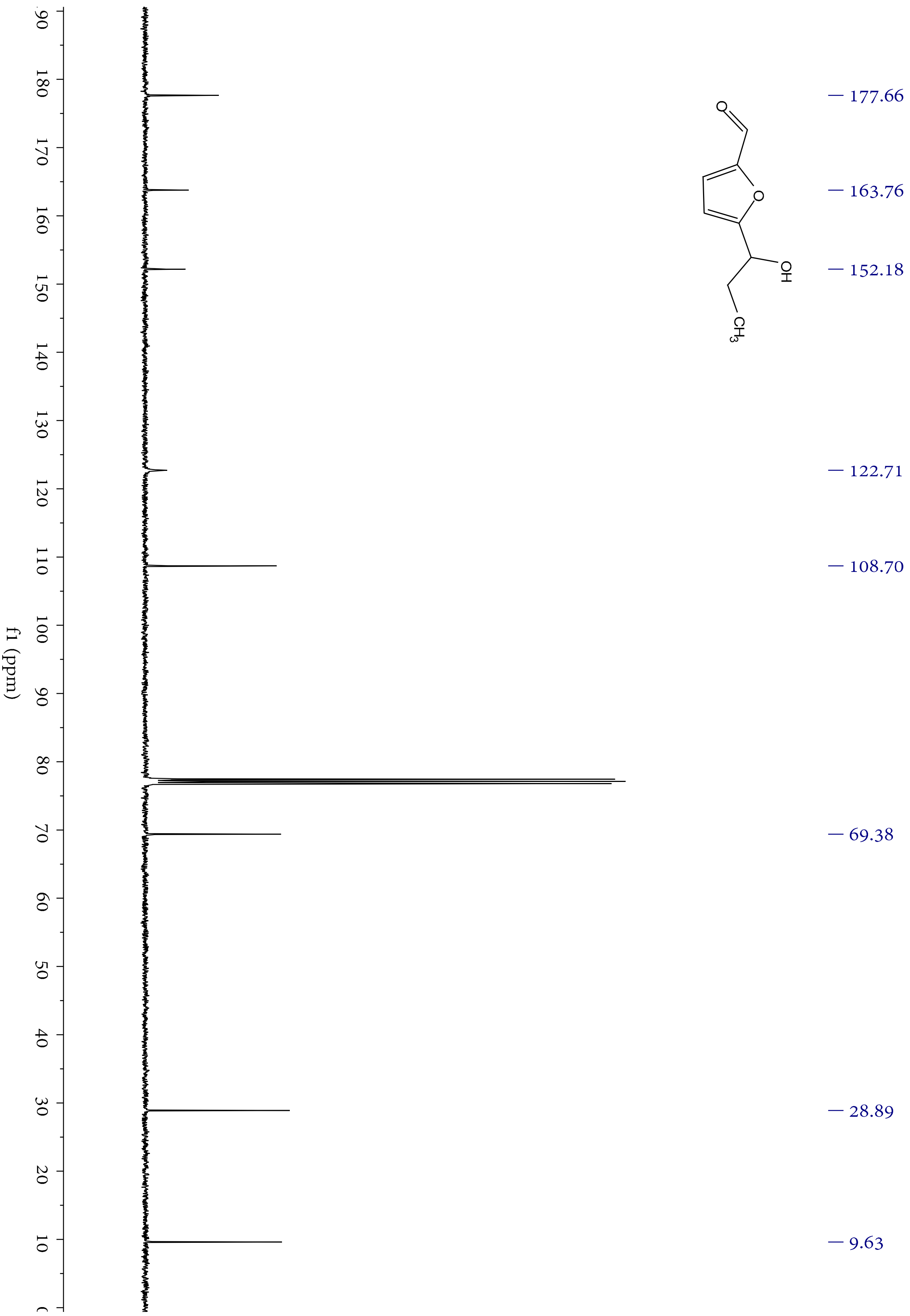

Figure S17. ^{13}C NMR spectrum of **17** recorded in $CDCl_3$ at 25°C (100 MHz)

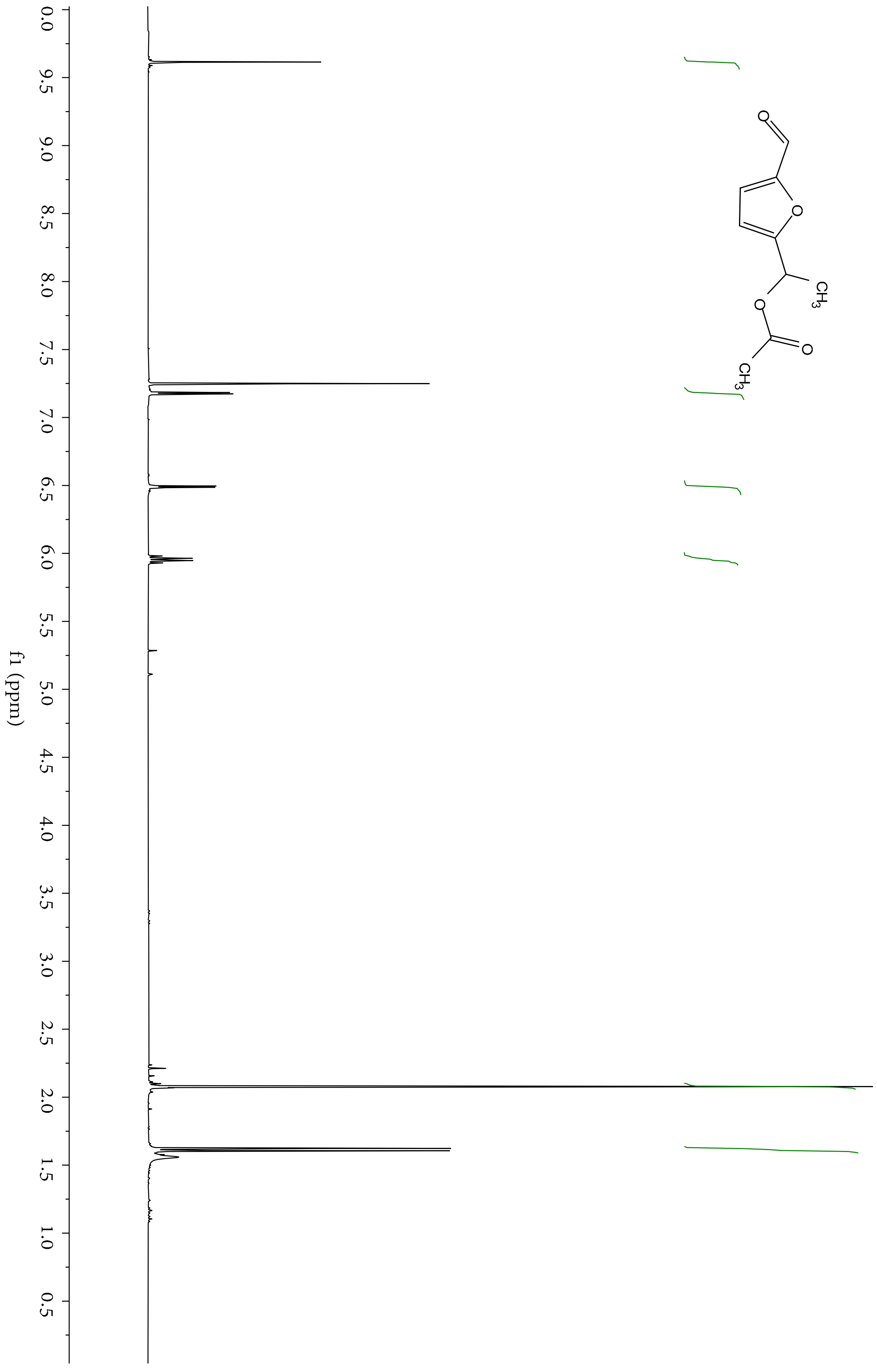

Figure S18. ^{1}H NMR spectrum of **18** recorded in $CDCl_3$ at 25ºC (400 MHz)

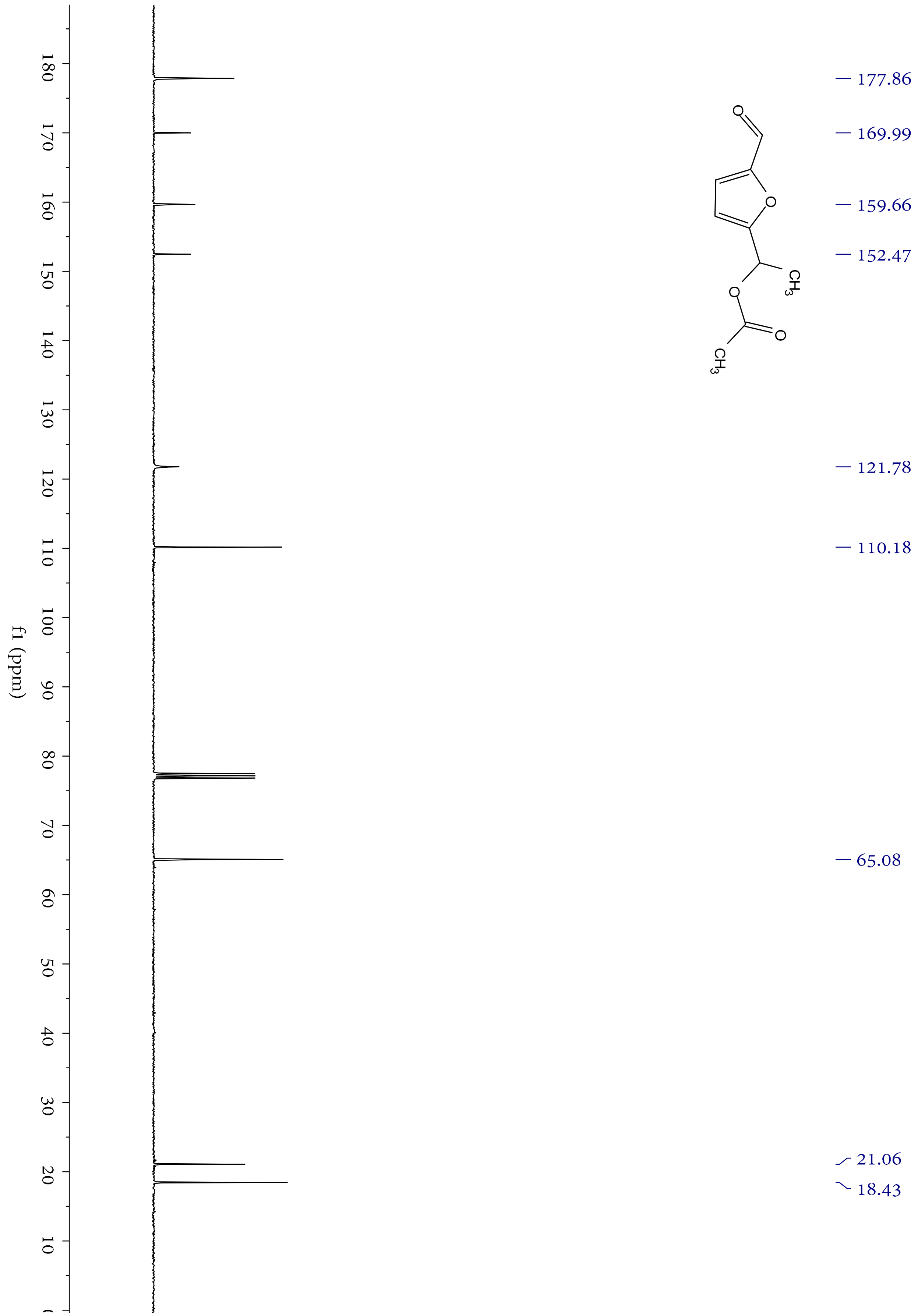

Figure S19. ^{13}C NMR spectrum of **18** recorded in $CDCl_3$ at 25°C (100 MHz)

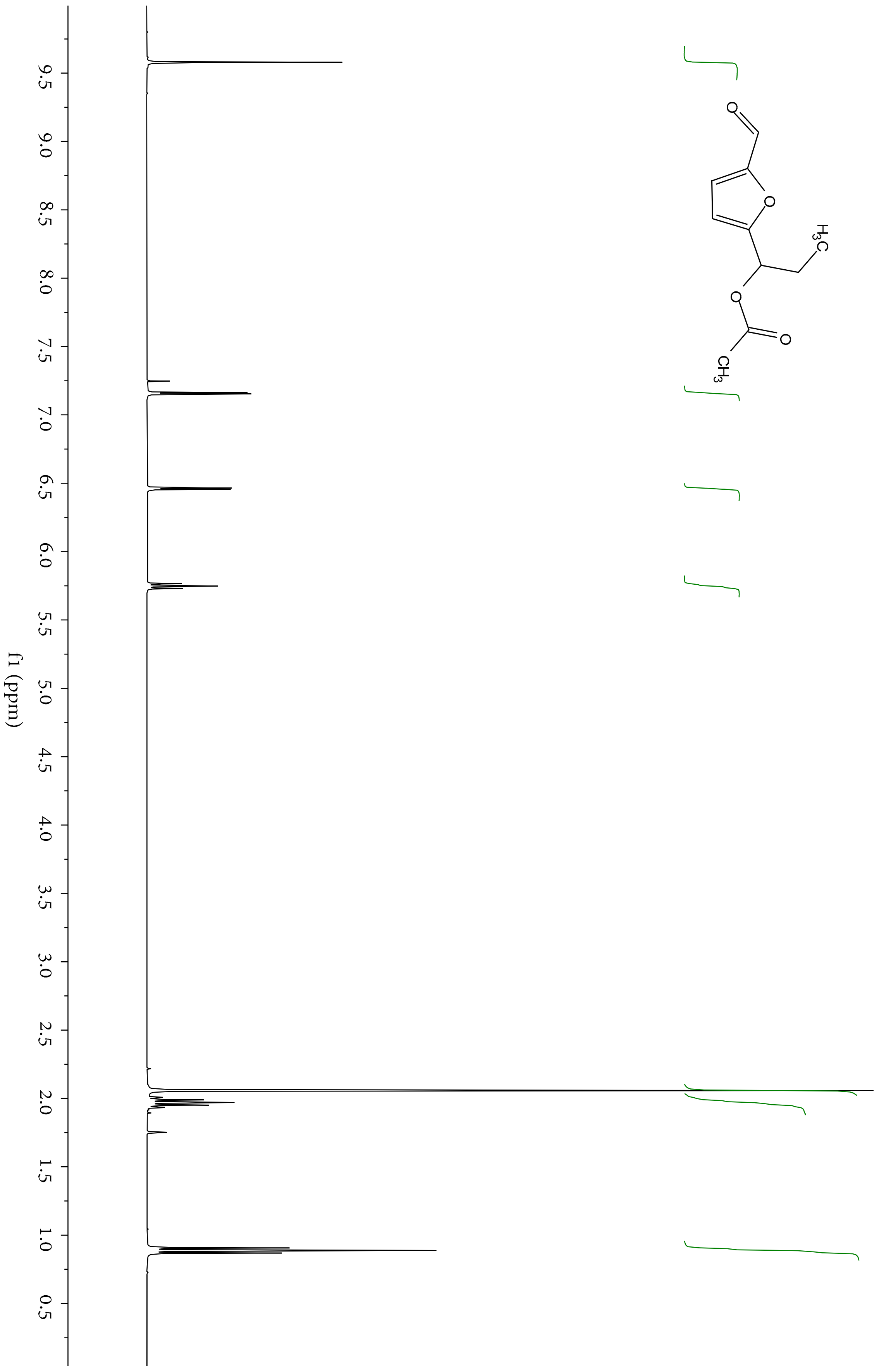

Figure S20. ^{1}H NMR spectrum of **19** recorded in $CDCl_3$ at 25°C (400 MHz)

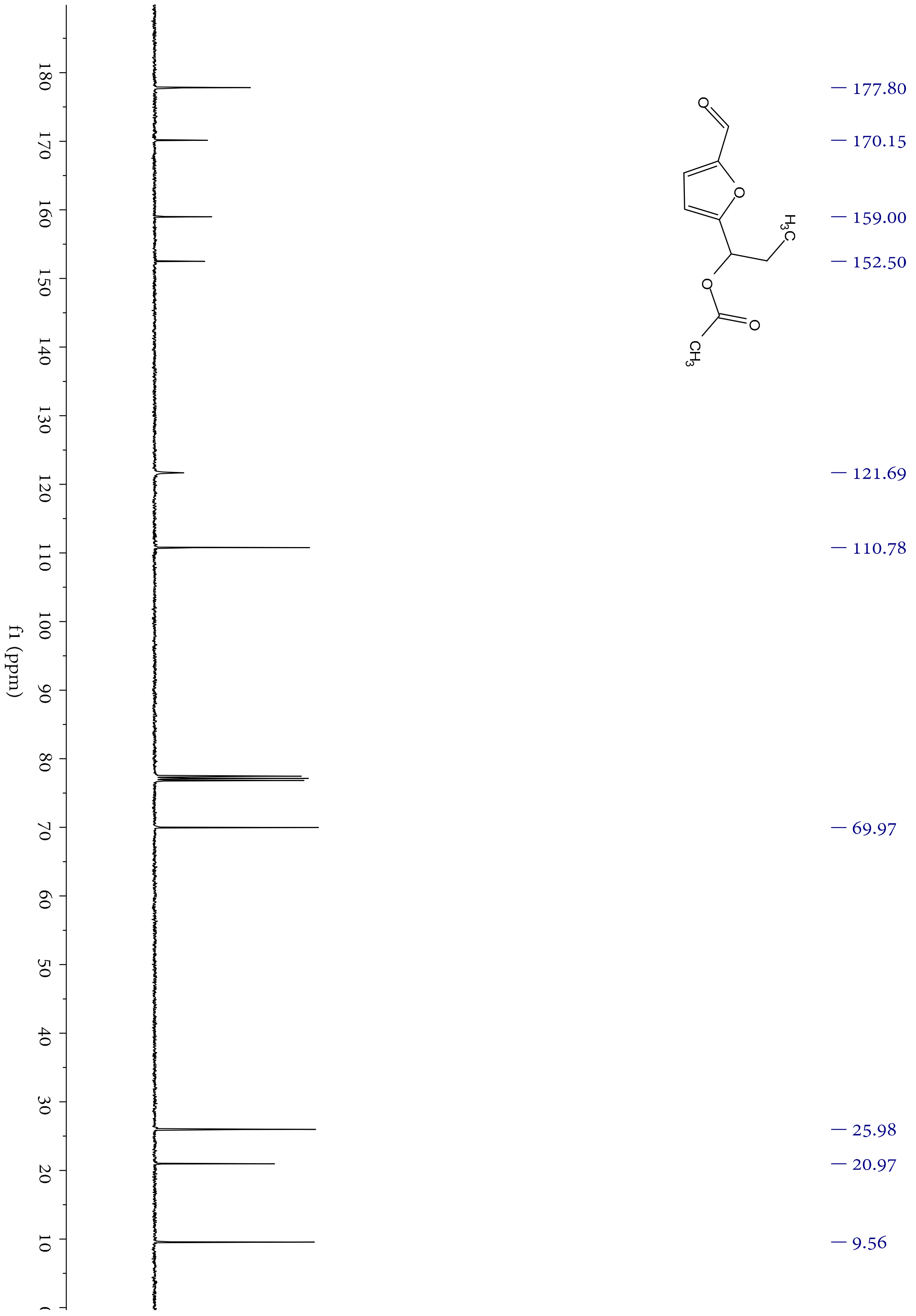

Figure S21. ^{13}C NMR spectrum of **19** recorded in $CDCl_3$ at 25°C (100 MHz)
